# Genome reconstructions indicate the partitioning of ecological functions inside a phytoplankton bloom in the Amundsen Sea, Antarctica

**DOI:** 10.3389/fmicb.2015.01090

**Published:** 2015-10-26

**Authors:** Tom O. Delmont, A. Murat Eren, Joseph H. Vineis, Anton F. Post

**Affiliations:** ^1^Josephine Bay Paul Center for Comparative Molecular Biology and Evolution, Marine Biological LaboratoryWoods Hole, MA, USA; ^2^Coastal Resources Center, Graduate School of Oceanography, University of Rhode IslandNarragansett, RI, USA

**Keywords:** Southern Ocean, Amundsen Sea Polynya, phytoplankton bloom, *Phaeocystis*, *Micromonas*, microbial communities, metagenomics, genome reconstruction

## Abstract

Antarctica polynyas support intense phytoplankton blooms, impacting their environment by a substantial depletion of inorganic carbon and nutrients. These blooms are dominated by the colony-forming haptophyte *Phaeocystis antarctica* and they are accompanied by a distinct bacterial population. Yet, the ecological role these bacteria may play in *P. antarctica* blooms awaits elucidation of their functional gene pool and of the geochemical activities they support. Here, we report on a metagenome (~160 million reads) analysis of the microbial community associated with a *P. antarctica* bloom event in the Amundsen Sea polynya (West Antarctica). Genomes of the most abundant Bacteroidetes and Proteobacteria populations have been reconstructed and a network analysis indicates a strong functional partitioning of these bacterial taxa. Three of them (SAR92, and members of the *Oceanospirillaceae* and *Cryomorphaceae*) are found in close association with *P. antarctica* colonies. Distinct features of their carbohydrate, nitrogen, sulfur and iron metabolisms may serve to support mutualistic relationships with *P. antarctica*. The SAR92 genome indicates a specialization in the degradation of fatty acids and dimethylsulfoniopropionate (compounds released by *P. antarctica)* into dimethyl sulfide, an aerosol precursor. The *Oceanospirillaceae* genome carries genes that may enhance algal physiology (cobalamin synthesis). Finally, the *Cryomorphaceae* genome is enriched in genes that function in cell or colony invasion. A novel pico-eukaryote, *Micromonas* related genome (19.6 Mb, ~94% completion) was also recovered. It contains the gene for an anti-freeze protein, which is lacking in *Micromonas* at lower latitudes. These draft genomes are representative for abundant microbial taxa across the Southern Ocean surface.

## Introduction

The Amundsen Sea polynya (ASP) covers a surface area of ~38,000 km^2^ during the height of austral summer and it is home to intense phytoplankton blooms. Chlorophyll *a* concentrations in the ASP peak in January and are 50% greater than blooms in other Antarctica polynyas (Arrigo and Van Dijken, 2003). Sediment cores demonstrated this polynya is >1000 years old (Kellogg and Kellogg, 1987; Hillenbrand et al., 2010) and its phytoplankton bloom, fueled by dissolved iron from glacier melt (Alderkamp et al., 2012), is currently dominated by large, spherically-shaped *Phaeocystis antarctica* colonies (Alderkamp et al., 2012; Mills et al., 2012; Kim et al., 2013; Delmont et al., 2014). Importantly, *P. antarctica* is capable of taking up twice as much CO_2_ per mole of phosphate removed than diatoms (Arrigo et al., 1999; Smith et al., 2003; Schoemann et al., 2005). It also produces copious amounts of dimethylsulfoniopropionate (DMSP) (DiTullio et al., 2000). Therefore, the substantial depletion of inorganic carbon to 100 ppm or less (Yager et al., 2012) coupled with high levels of dimethylsulfide (DMS) (Tortell et al., 2012) in the ASP illustrate the immediate effects of this phytoplankton bloom on the regional carbon, nutrient and sulfur cycles.

This intense *Phaeocystis* productivity generally carries on for about a 3 month period, spanning most of the austral summer (Arrigo and Van Dijken, 2003). The blooms are accompanied by a distinct (photo) heterotrophic community. This includes a small, numerically insignificant population of Archaea and a diverse bacterial community (Kim et al., 2013; Delmont et al., 2014). Yet, the ecology and functioning of the bacterial populations evolving in this system is still poorly understood. We know that bacterial evenness is stable and unusually low in the polynya surface in comparison to that in surrounding waters, with four taxa (SAR92, *Oceanospirillum, Polaribacter* and *Pelagibacter*) accounting for about 75% of the cells (Kim et al., 2013; Delmont et al., 2014; Williams et al., 2014). Sensitive partitioning of 16S rRNA sequences (oligotyping) suggests that these populations are dominated by a single genotype (Delmont et al., 2014). Some of these (SAR92, *Oceanospirillum* and less dominant taxa such as members of the *Cryomorphaceae*) are preferentially associated with *Phaeocystis* colonies (Delmont et al., 2014) and display higher heterotrophic activity than the free-living bacteria (Williams et al., 2014). These studies did not resolve whether bacterial populations were attached at the surface of algal colonies or resided inside colony matrix. Nonetheless, these observations support the concept of physically delineated bacterial niches and suggest functional interactions between the alga and specialized bacteria. It also suggests that physiological and ecological functions carried out by heterotrophs are not evenly distributed. E.g., the cycling of carbon and nutrients by bacteria inside a *Phaeocystis* colony is likely very different from those contributed by free-living bacteria.

*Phaeocystis antarctica* blooms in the ASP shape bacterial community structures and their genotype composition is maintained over time and space (Delmont et al., 2014). Efforts have been made to isolate and characterize keystone bacterial species in the Southern Ocean (Bowman et al., 1997). However, most bacterial taxa (including those associated with *Phaeocystis* species) are recalcitrant to cultivation (Janse et al., 2000), limiting our ability to study their contribution to carbon and nutrient cycles of the ASP. Metagenomic approaches have been used to determine the functional potential of bacteria in Southern Ocean habitats (Wilkins et al., 2013a,b). These approaches can link taxonomy and function through the assembly of dominant genetic structures (Tyson et al., 2004; Grzymski et al., 2012). On one side, metagenomic assemblies of complex eukaryotic genomes are challenging due to the occurrence of repeat DNA regions (Richard et al., 2008) that cannot be overcome with most sequencing technologies. This technological limitation might affect the effective assembly of *P. antarctica* and diatom genomes directly from bloom events. On the other hand, the presence of few, highly abundant bacterial taxa in surface waters of the ASP provides an opportunity to determine the genome content of the dominant bacterial taxa, be they free-living or associated with *P. antarctica* colonies.

Here, we use environmental DNA extracted from a 0.2 to 20 μm filtered plankton size fraction and deeply sequence and assemble genetic structures to determine physiological and metabolic contributions of microbial taxa of a centrally located sample of this productive bloom. This sequencing effort exceeds the combined metagenomic data previously generated for other Southern Ocean locales and resulted in the assembly of several microbial genomes. Novel draft genomes were affiliated to six heterotrophic bacterial taxa (including SAR92, *Polaribacter, Oceanospirillaceae*, and *Cryomorphaceae*) and an estimated 94% of a novel *Micromonas* genome (19.6 Mb recovered). For *Phaeocystis* (the most abundant phytoplankter) the lack of significant genome assembly warranted the use of RNA-seq derived transcriptome from a culture strain (Delmont et al., under submission) to infer its functional potential. We report here on a total of 41,805 protein coding sequences with 5278 distinct functions determined from the draft genomes. Gene functions were used to generate a network that identified coupled ecosystem functions between dominant primary producers and the bacterial community. Finally, we exploited publically available metagenomes to determine in which extent these draft genomes are representative for abundant microbial taxa across the Southern Ocean.

## Results

### General

Unassembled paired-end reads of the metagenome of the microbial community associated with a *P. antarctica* surface bloom in the central ASP were analyzed using protein and ribosomal reference databases within MG-RAST (Meyer et al., 2008). Taxonomy assignments (Figure S1) distributed the reads over members of the Eukaryota (0.27 ± 0.24 of total reads), Bacteria (0.73 ± 0.24) and Archaea (0.06 ± 0.03), with variation determined by the choice of the reference protein or ribosomal database. The eukaryotic fraction was dominated by diatoms, Chlorophytes and Haptophytes, while Bacteroidetes and Proteobacteria related organisms dominated the bacterial fraction. These findings were in close agreement with the composition of the microbial community that was independently determined (16S rRNA gene survey) for the same sample as well as for various other locations inside the *P. antarctica* bloom over a 3 week period (Delmont et al., 2014).

From a total of 159.3 million paired-end and mate-pair sequence reads we assembled longer genetic structures of the ASP metagenome. Approximately 30% of the reads assembled into 56,805 scaffolds >1 kb for a total of 154.8 Mb. 92% of these scaffolds were < 5 kb with some of the most abundant ones consisting of short, high repeat regions of eukaryotic genomes. On the other side of the assembly spectrum, the 1500 longest scaffolds ranged from 12 to 333 kb in length with 12-743X in coverage and GC contents that ranged from 27 to 65%.

### A pipeline for draft genome assembly

**Figure 1:**
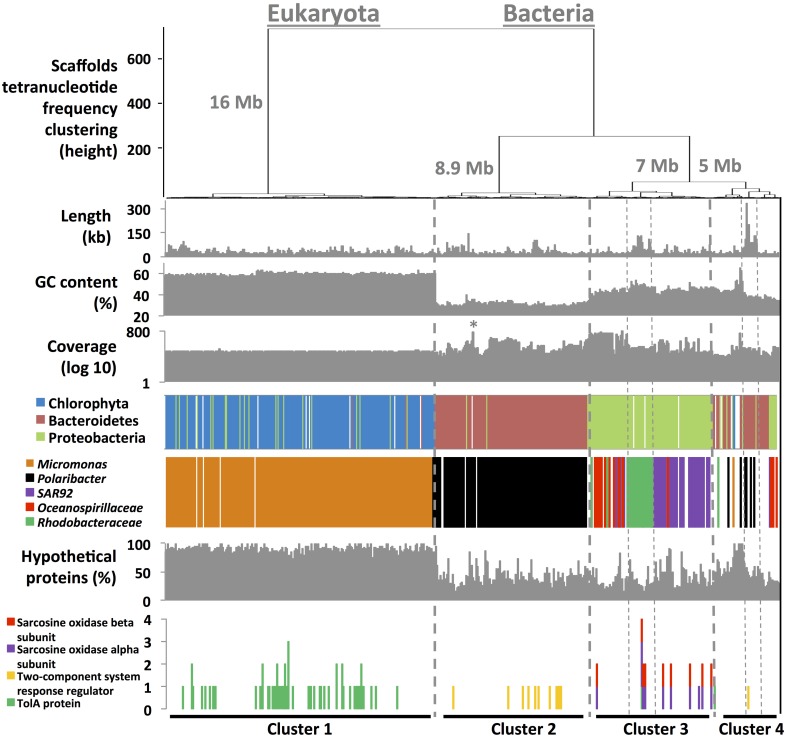
**Hierarchical clustering (Euclidean distance metric) of 1500 scaffolds (11.9–332.7 kb in length) based on their tetranucleotide frequency profiles**. Seven informative layers were added below to the clustering tree. Percentage of hypothetical proteins and the occurrence of specific functions were accessed using RAST (Aziz et al., 2008). Taxonomical affiliation was inferred using phymmBL (Brady and Salzberg, 2009). The ^*^ in coverage value layer represents *Phaeocystis antarctica* chloroplast scaffolds.

A simple four-step method was designed to bin assembled metagenome fragments into draft genomes without requiring a reference database. First, we determined assembly characteristics (%GC, length, tetranucleotide frequency, coverage) for each scaffold using in-house developed scripts. In the two next steps, we inferred scaffold taxonomy using phymmBL (Brady and Salzberg, 2009) and used RAST (Aziz et al., 2008) for functional annotation. A total of 37,143 genes and 5116 functions were detected from the 1500 longest scaffolds (37 Mb of assembly). 87% of these scaffolds had blast hits with average blast e-value score of < 10^−108^, which permitted taxonomy inference with a reasonable degree of certainty. Finally, we generated dendrograms that cluster scaffolds based on tetranucleotide frequencies (Euclidean distances). Dendrograms were then coupled to scaffold characteristics (step 1) and nearest neighbor taxonomy (step 2) to define single taxon clusters (Figure 1). Scaffolds clustered into four major tetranucleotide frequency clusters. Those that fell into Cluster 1 (affiliated with pico-eukaryote algae, see below) were characterized by low coverage (50 ± 4X), a high %GC (59 ± 2%) and a relatively high percentage of hypothetical proteins (82 ± 10%). Scaffolds from Cluster 2 had a low %GC content and subdivided into three groups with distinctly different coverage (Figure 1), each of them with *Flavobacteria* taxonomy that had *Polaribacter* as it closest relative. This cluster also contained low %GC scaffolds of organelles, both chloroplast and mitochondria, which were analyzed separately. Cluster 3 represented three groups affiliated to *Oceanospirillaceae* (high coverage), SAR92 (Gammaproteobacteria) and the *Rhodobacteraceae* (Alphaproteobacteria), all of them with similar %GC, distinct from those in clusters 1 and 2. Cluster 4 contained scaffolds with different taxonomical affiliations, including a well assembled bacterial group that affiliated with known members of the *Cryomorphaceae* and *Flavobacteraceae*. The clusters do not only have distinct taxonomical affiliations, they also harbor taxon-specific functions: e.g., Cluster 1 was the sole cluster with genes that encode TolA related proteins typically found in eukaryotes (Levengood-Freyermuth et al., 1993). A two-component system regulator implicated in the response to environmental cues in Bacteroidetes (West and Stock, 2001) was found in Cluster 2 only. Paired functions typical of Proteobacteria (e.g., sarcosine oxidase subunits) were observed on the same scaffolds in Cluster 3. Finally, we targeted subsets of the metagenome data to perform additional assemblies and tetranucleotide frequency ordinations to yield 3553 scaffolds of >6 kb in length, contributing to the completion of the draft genomes (see Appendix and Figures S2–S6).

### Draft genomes for dominant taxa of the *P. antarctica* bloom

*Phaeocystis* dominated this bloom event, yet its genome was not part of the assembly clusters described above. Clearly, challenging features like the occurrence of DNA repeats and the lack of reference genome prevented a meaningful assembly and downstream analysis of an environmental *P. antarctica* genome. Nonetheless, we could detect *Phaeocystis* using alternative approaches. First, MG-RAST identified 10,680 of 127,009 rRNA reads in our data set as *Phaeocystis* ribosomal RNA with high confidence (average *e*-value of < 10^−65^ against M5RNA data base). Second, the *P. antarctica* chloroplast (107 kb in length, NCBI accession JN117275.2) was fully recovered with >700X coverage, further confirming its dominance in the ASP metagenome. Aside from short contigs of *Phaeocystis* (up to 70% of the metagenome), we obtained a total of 10 draft genomes for the ASP sample and summarized their general features in Table 1. We estimated their relative abundance in the targeted phytoplankton size fraction by mapping quality-filtered reads to each draft genome using a stringent mapping criterion (>97% sequence identity over the entire read length) and determined their taxonomical affiliation and gene content using RAST.

**Table 1:** **General features related to 10 draft genomes determined from our metagenomic assembly and bioinformatics approach**.

**General features**	**Best taxonomical hit (RAST collection)**	**Reads in assembly**	**Scaffolds length**	**Total length (Mb)**	**Number of scaffolds**	**GC content**	**Coverage**	**Fraction of the metagenome**	**Number of coding sequences**	**Number of different functions**	**Different tRNA**	**Fraction of hypothetical proteins**
***PHAEOCYSTIS ANTARCTICA***												
Chloroplast (JNl17275.2)	/	/	/	0.1	/	35.5%	740X	0.28%	133	117	20	9.0%
**CANDIDATUS CHLOROPHYTA**												
Micromonas ASP10-01a	/	159 millions	>6kb	19.6	1071	58.9%	49X	5.63%	22,252	1813	17	82.1%
**CANDIDATUS PROTEOBACTERIA**												
Oceanospirillaceae ASP10-02a	Marinomonas	20 millions	>10kb	2.5	80	45.0%	732X	7.93%	2343	1672	18	16.6%
SAR92 ASP10-03a	SAR92	159 millions	>10kb	3.2	142	45.7%	80X	1.11%	2892	1474	12	22.9%
Rhodobacteraceae ASP10-04a	Roseobacter	159 millions	>10kb	2.8	61	48.1%	105X	1.27%	2748	1802	18	17.2%
**CANDIDATUS BACTEROIDETES**												
Cryomorphaceae ASP10-05a	Pedobacter saltans	159 millions	>10kb	2.9	44	38.2%	99X	1.03%	2450	1322	19	33.7%
Polaribacter ASP10-06a	Polaribacter irgensii	40 millions	>10kb	2.9	165	34.4%	272X	3.42%	2400	1452	15	18.7%
Polaribacter ASP10-07a	Polaribacter irgensii	159 millions	>10kb	1.8	110	32.0%	108X	0.84%	1567	902	10	20.1%
Polaribacter ASP10-08a	Polaribacter irgensii	159 millions	>10kb	1.9	70	29.9%	56X	0.46%	1598	1097	11	14.5%
Flavobacteriaceae ASP10-09a	Tenacibaculum	159 millions	>10kb	2.5	71	30.3%	60X	0.65%	2100	1450	15	17.9%
Flavobacteriaceae ASP10-10a	Flavobacteriales bacterium	159 millions	>10kb	1.2	55	32.5%	83X	0.43%	1296	641	20	32.9%
Total for the 10 draft genomes				41.3	1459			26%	41,779	5278		

*Their percentage of the metagenome was determined using CLC software (version 6), the entire datasets and a mapping stringency of 97% (full length required). Finally, functional information is derived from RAST annotation (26)*.

**Figure 2:**
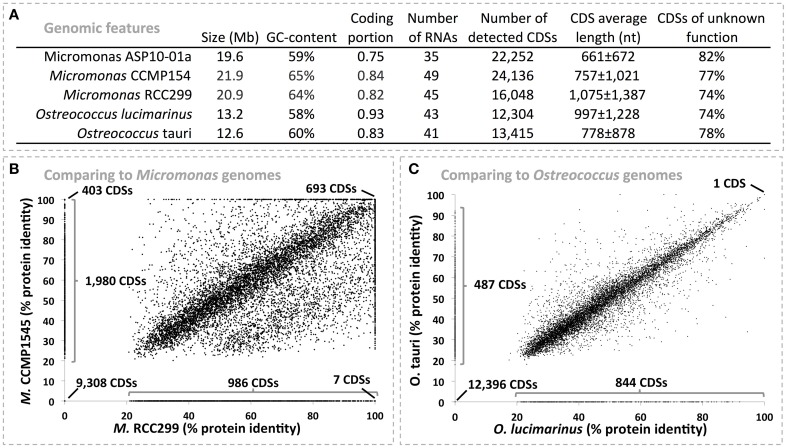
**(A)** Displays general functional features of five algal genomes based on RAST annotation. **(B,C)** represents protein identity scores between the 22,278 protein coding sequences (CDSs) from the *Micromonas* ASP10-01a draft genome recovered from the ASP and those detected in *Micromonas* **(B)** and *Ostreococcus* **(C)** available genomes. Scores were determined using the RAST genome comparison tool (Aziz et al., 2008). We performed two comparisons per panel, which provided two distinct identity scores for each of the *Micromonas* ASP10-01a proteins (one in each axis). Note that the score was set to 0 when a protein from *Micromonas* ASP10-01a had no match with proteins from the compared genome.

We recovered a Chlorophyte draft genome that was 19.6 Mb in length with 22,278 detected protein coding sequences (CDSs) and accounted for 5.63% of the metagenomic data. 93.8% of the total genome was recovered based on a scaffold length logarithmic regression curve estimate (*R*^2^ > 0.997, see Figure S7). Using phymmBL we identified this draft genome as belonging to a member of the *Micromonas* (Figure 1), a genus from the order *Mamiellales*, which includes picophytoplankton species. This taxonomic affiliation was further supported by MG-RAST with the identification of 2883 rRNA reads as *Micromonas* (average *e*-value of 10^−61^ against M5RNA database). There are four publically available *Mamiellales* genomes: they correspond to *Micromonas* strains CCMP1545 and RCC299 along with *Ostreococcus tauri* and *O. lucimarinus*, organisms isolated from marine habitats at lower latitudes (Worden et al., 2009). We compared the picoeukaryote draft genome to these genomes at the predicted protein level to better establish its taxonomic affiliation and identify unique features of its functional repertoire. Overall, the CDSs of picoeukaryote draft genome were similar in number and length to *Micromonas* genomes but had a GC-content that was more similar to the *Ostreococcus* genomes (Figure 2A). It also harbored a lower density of coding sequences and higher proportion of hypothetical genes relative to the four reference genomes, possibly due to its draft stage. Based on protein identity values, the draft genome shared more CDSs with the *Micromonas* genomes than with the *Ostreococcus* genomes (Figures 2B,C). CDSs identical in the ASP picoeukaryote draft genome and the two *Micromonas* genomes include core genome functions related to ferredoxin synthesis, photosystem biogenesis, haem formation, cell division and nutrient transport. Overall, CDSs were 35.6 ± 37%, 30 ± 35%, 20 ± 26%, and 19 ± 25% identical when comparing the ASP draft genome to the *Micromonas* CCMP1545, *Micromonas* RCC299, *O. lucimarinus* and *O. tauri* genomes respectively. Based on the findings above we propose that the ASP picoeukaryote draft genome belongs to a novel Antarctic *Micromonas* species and will refer to it as *Micromonas* ASP10-01a in the following paragraphs. We do note that a large fraction of CDSs (*n* = 9011) in the ASP *Micromonas* draft genome were not detected (CDS identity < 20%) in any of the available *Mamiellales* genomes. Of these, the vast majority (98.1%) was annotated as hypothetical proteins.

Annotation of putative genes in the *Micromonas* ASP10-01a draft genome was difficult to achieve given the current lack of knowledge on eukaryotic functionalities (Figure 1), leading to the detection of only 1535 different functions. Several functions were present in higher copy number in *Micromonas* ASP10-01a (compared to the reference *Mamiellales* genomes), including those that encode ankyrin repeat proteins (structural motifs implied in attaching membrane proteins to the cytoskeleton; *n* = 65). Another function present with high copy number was related to enoyl-CoA hydratase, suggesting an enhanced role for fatty acid metabolism in the Antarctic *Micromonas*. We also used the totality of deduced gene functions annotated across the five *Mamiellales* genomes (*n* = 3086) to create a functional network (Figure S8). Although, 945 functions were shared between the five genomes, we identified 200 functions that were unique to *Micromonas* ASP10-01a and included a novel anti-freeze protein. Best blastx hit against the Genbank nr database identified the anti-freeze protein of a psychrophilic fungus (*Typhula ishikariensis*) with a protein sequence identity of 36%.

Annotation of bacterial draft genomes was more straightforward (Table 1), yet their novelty and general lack of 16S rRNA genes prevented sensitive taxonomy assignment. Therefore, we assigned most of these draft genomes at the family level within Proteobacteria and Bacteroidetes and so ensure their correct affiliation. A draft genome for a member of the *Oceanospirillaceae* (Gammaproteobacteria) incorporated about 8% of the metagenomic reads in its assembly and a total of 2343 putative CDSs were identified. The most similar organism in the RAST database was *Marinomonas* sp. MWYL1 with a protein identity score of 35 ± 25%. A second draft genome was identified as a member of the SAR92 group as it was most closely related to the temperate SAR92 strain HTCC2207, the only isolate for which a genome sequence was determined (Stingl et al., 2007). The two genomes shared a protein identity score of 46 ± 32%, with 743 putative CDSs detected in the ASP SAR92 draft genome only. A third draft genome was derived from an alphaproteobacterium with >3000 CDSs identified and a total of 1802 annotated functions. The genome most closely related in RAST database was that of *Roseobacter sp. GAI101* (protein identity of 45 ± 29%), a member of the *Rhodobacteraceae*.

The *Cryomorphaceae* draft genome had no close relative in the RAST database. The closest organism to this genome was *Pedobacter saltans* 12145 (protein identity score of 26 ± 24%). We also searched the 812 bases 16S rRNA gene fragment we found in this draft genome against NCBI's nr database and further confirmed its affiliation to the *Flavobacteria* class. The closest entry in NCBI's refseq genomic database was *Fluviicola taffensis* (query cover of 99% and identity of 87%), suggesting the presence of a new type of organism within the *Cryomorphaceae* family. After including the *Fluviicola taffensis* genome into RAST, we determined that the two organisms possess a protein identity score of 32 ± 24% only. Finally, the *Flavobacteriaceae* cluster provided 5 distinct groups (Figure S2) affiliated with *Polaribacter irgensii* 23-P (3 draft genomes with protein sequence identity of 69 ± 36%, 61 ± 34%, and 59 ± 27%), *Tenacibaculum* sp. MED152 (protein identity of 63 ± 29.7%) and *Flavobacteriales bacterium* HTCC2170 (protein identity of 30 ± 31%). However, three of these draft genomes were likely incomplete with an overall length ranging from 1.2 to 1.9 Mb. Therefore, only draft genomes related to *Tenacibaculum* (2.5 Mb) and one of the *Polaribacter* (2.9 Mb) were part of further analyses described in the following sections. Note that *Tenacibaculum* was not detected in our high throughput 16S-V6 rRNA screening of the same bloom event but it is phylogenetically closely related to *Polaribacter* (Yoon et al., 2006). *Polaribacter* was identified as an abundant taxon in the ASP microbial community (Delmont et al., 2014). Because of this uncertainty, we conservatively assigned the genome as *Flavobacteriaceae* ASP10-09a.

### Functional diversity indicates a distinct role of each dominant bacterial population

We effectively reconstructed 7 draft genomes (three Proteobacteria, three Bacteroidetes, and one pico-eukaryote) from combined mate-pair and paired-end reads of the ASP metagenome. We acknowledge that genomes are likely incomplete (tRNAs suggest completions of 50–100%) but their length (2.5–3.2 Mbp) and number of detected genes (2100–2892) indicates high recovery rates. This enabled us to interrogate aspects of physiological functions and life styles that underpin their ecological roles within the ASP microbial community. The draft aspect of these genomes prevented conclusions regarding intact metabolic pathways as not always were all genes identified. Nonetheless, we observed clear differences: genes encoding the Entner-Doudoroff pathway were detected in the Proteobacteria genomes only whereas genes encoding the pentose phosphate pathway were specific to the *Rhodobacteraceae* ASP10-04a genome. Following sections describe more specific differences at the subcategory and functional level.

**Figure 3:**
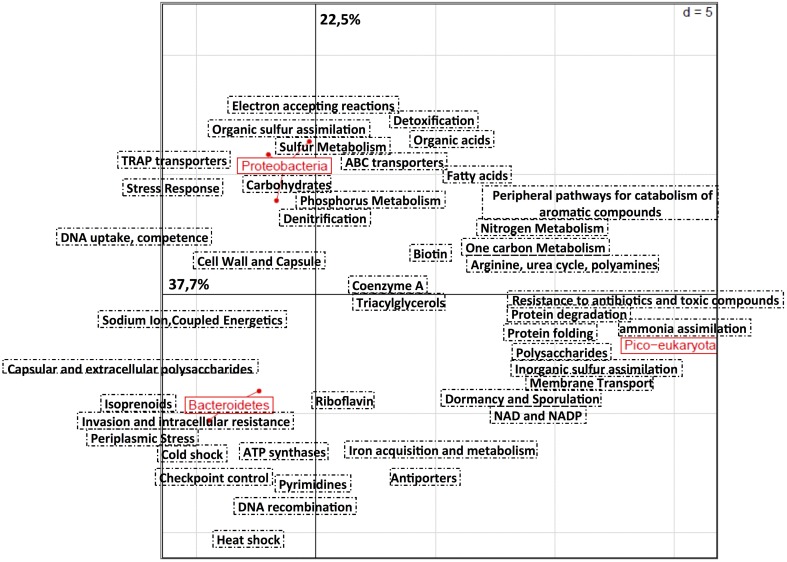
**Principal component analysis based on the relative distribution of 105 functional subsystems in seven draft genomes recovered from the ASP**. Annotation was done using RAST (Aziz et al., 2008). The analysis was performed using R software and the Ade4TkGUI package (Thioulouse et al., 1997). Proteobacteria includes *Oceanospirillaceae* ASP10-02a, SAR92 ASP10-03a, and *Rhodobacteraceae* ASP10-04a. Bacteroidetes includes *Cryomorphaceae* ASP10-05a, *Polaribacter* ASP10-06a, and *Flavobacteriaceae* ASP10-09a. Finally, *Micromonas* ASP10-1a is the only pico-eukaryota representative.

As a first approach we performed a principal component analysis based on the relative distribution of 105 subcategories defined by the SEED annotation on RAST (Figure 3). Draft genomes clustered according to their taxonomic affiliation, emphasizing a strong link between function and taxonomy. Subcategories for protein metabolism (protein folding, biosynthesis and degradation), inorganic sulfur assimilation and ammonia assimilation (e.g., multiple ammonium transporters), membrane transport, and polysaccharides distinguished the draft genome of *Micromonas* from bacterial genomes. The abundance of genes related to periplasmic stress responses, invasion and intracellular resistance, DNA recombination, and ATP synthase were distinguishing features of the Bacteroidetes genomes. Interestingly, they also contained multiple genes implied in thermal shock responses (e.g., cold shock protein CspA). Finally, Proteobacteria genomes showed a prevalence of genes involved in environmental stress response and detoxification, denitrification, sulfur, and phosphorus metabolism, organic sulfur assimilation, TRAP, and ABC transporters, carbohydrate and electron accepting reactions.

In addition, we attempted to identify statistical difference between functions of the bacterial genomes (*n* = 4253). Using STAMP (Parks and Beiko, 2010) we applied a *t*-test (equal variance) in order to discriminate functions differentially present between taxa (Bacteroidetes vs. Proteobacteria). We determined that a total of 354 functions distinguished between these taxa (*p* < 0.05, Dataset S1). We also compared differences in gene functions between free-living taxa (*Rhodobacteraceae*; *Polaribacter*; *Flavobacteriaceae*) and particle associated taxa (*Oceanospirillaceae*; SAR92; *Cryomorphaceae*) as determined from a 16S rRNA gene survey (Delmont et al., 2014). In this comparison a total of 24 functions were differentiated between the two lifestyles (*p* < 0.05, Dataset S1). Interestingly, a multimodular transpeptidase-transglycosylase (also named penicillin-binding protein) was only detected in genomes of particle-associated bacteria. This protein is involved in peptidoglycan synthesis, protein-protein attachment and antibiotic resistance (Goffin and Ghuysen, 1998). Other functions characteristic to genomes of particle-associated bacteria include an aquaporin Z (involved in osmoregulation), a protein motif for binding to peptidoglycans and the universal stress protein UspA. However, their possible roles in offsetting antimicrobial activity of *Phaeocystis*, in attachment to its surface or in living inside colonies await targeted experiments.

**Figure 4:**
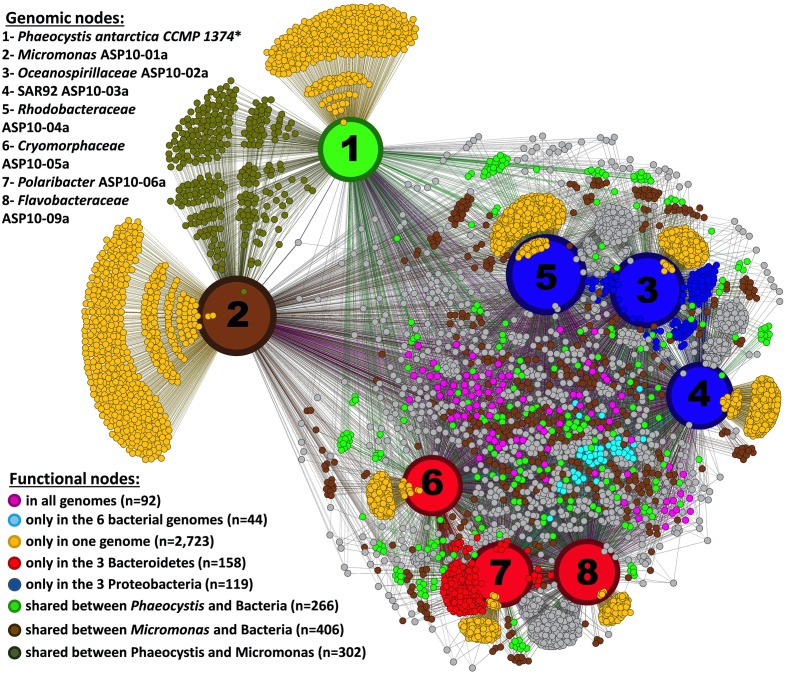
Network connecting seven draft genomes recovered from the ASP plus the transcriptome of *Phaeocystis antarctica* strain CCMP 1374 (^*^based on 33,153 contigs screened from the assembly of 99,805,691 HiSeq and 2,492,614 pyrosequencing RNA-seq reads) and their annotated functions from RAST (Aziz et al., 2008) (a total of 5590 functions). The network was performed using Gephi (Bastian et al., 2009) and Force Atlas 2.

In a more global analysis, we also investigated differences between the dominant photosynthetic (*Phaeocystis, Micromonas*) and heterotrophic (Proteobacteria, Bacteroidetes) components of the ASP bloom. Since metagenome assembly of *Phaeocystis* reads did not yield scaffolds long enough to be informative here, we included a RAST annotated transcriptome of the *P. antarctica* strain CCMP 1374 (Delmont et al., under submission) in this analysis. The 5590 different functions detected in the bacterial and *Micromonas* draft genomes and the *P. antarctica* transcriptome were used to generate a functional network that identified both shared and unique (and possibly complementary) functions in the plankton community of the ASP (Figure 4). Only 92 functions were shared among all the organisms. This low number reflects the draft stage of recovered genomes, their distant taxonomical affiliation and annotation limitations. Some functions were shared among the members of a taxonomic group: e.g., functions found only in the three Bacteroidetes (*n* = 158) or in the three Proteobacteria (*n* = 119). In addition, the genomes of these organisms encompass a total of 2723 unique functions. In the following sections we describe the occurrence of functions related to the cycles of nitrogen, sulfur, carbon, and iron, as well as various other genes related to outer membrane proteins, vitamin synthesis and light harvesting (Table 2). Note that we confirmed the functional inference of genes described below using blastx against the *nr* database in NCBI (see Appendix and Dataset S2).

**Table 2:** **Compilation of functional features detected in one or more of the seven draft genomes recovered from the Amundsen polynya with a length >2.5 Mb (*Oceanospirillaceae* ASP10-02a; SAR92 ASP10-03a; *Rhodobacteraceae* ASP10-04a; *Cryomorphaceae* ASP10-05a; *Polaribacter* ASP10-06a; *Flavobacteriaceae* ASP10-09a and *Micromonas* ASP10-01a)**.

	**Proteobacteria**			**Bacteroidetes**			**Chlorophyta**	**Haptophyta**
	**Oceanospffillaceae**	**SAR92**	**Rhodobacteraceae**	**Cryomorphaceae**	**Polaribacter**	**Flavobacteriaceae**	**Micromonas**	**Phaeocystis**
**NITROGEN METABOLISM**								
Nitrite to Ammonia^**^	No	No	No	Yes^**^	Yes^**^	Yes^**^	Yes^**^	Yes^**^
Nitrate/Nitrite transporters	Yes^*^	Yes^*^	No	No	No	No	Yes^*^	Yes^*^
Ammonium transporter	Yes^*^	Yes^*^	Yes^*^	No	No	No	Yes^*^	Yes^*^
Ammonia and Glutamate to Glutamine^**^	Yes^**^	Yes^**^	Yes^**^	Yes^**^	Yes^**^	Yes^**^	Yes^**^	Yes^**^
Allantoin Utilization	No	No	Yes^*^	No	No	No	No	No
**SULFUR METABOLISM**								
Sulfate to Sulfide^**^	Yes^**^	No	No	No	No	No	Yes^**^	No
Sulfur oxidation (SOX system)	No	No	Yes^*^	No	No	No	No	No
Thioredoxin reductase^**^	Yes^**^	Yes^**^	No	Yes^**^	Yes^**^	Yes^**^	Yes^**^	Yes^**^
DMSP acyl CoA transferase (DddD)	No	Yes^*^	No	No	No	No	No	No
DMSP demethylase (DmdA)	Yes^*^	No	Yes^*^	No	No	No	No	No
Arylsu Ifatase	No	Yes^*^	No	No	Yes^*^	Yes^*^	Yes^*^	Yes^*^
**CARBOHYDRATE METABOLISM**								
Carbonic Acid to C02^**^	Yes^**^	Yes^**^	Yes^**^	Yes^**^	Yes^**^	Yes^**^	No	Yes^**^
Carbon monoxide dehydrogenase	No	Yes^*^	Yes^*^	No	No	No	No	No
Pyruvate to AcetylCoA^**^	No	Yes^**^	No	No	No	No	Yes^**^	Yes^**^
Pyruvate metabolism I^**^	Yes^**^	No	Yes^**^	Yes^**^	Yes^**^	Yes^**^	Yes^**^	Yes^**^
Pectin degradation	No	Yes^*^	No	No	No	No	No	No
**IRON ACQUISITION**								
Protoporphyrin to Heme	Yes^*^	Yes^*^	Yes^*^	Yes^*^	Yes^*^	No	Yes^*^	Yes^*^
Ferro-chelatase	Yes^*^	Yes^*^	Yes^*^	Yes^*^	Yes^*^	No	Yes^*^	No
Siderophore biosynthesis	No	No	No	Yes^*^	Yes^*^	Yes^*^	No	No
Ferric siderophore transport system	No	Yes^*^	No	No	No	No	No	No
Ferric iron ABC transporter	Yes^*^	No	Yes^*^	Yes^*^	No	No	No	No
Ferrous iron transport protein B	No	No	No	No	Yes^*^	Yes^*^	No	No
Iron(lIl) ABC transporter, ATP-binding protein	No	No	No	No	Yes^*^	Yes^*^	No	Yes^*^
Outer membrane hemin receptor	No	No	No	No	Yes^*^	Yes^*^	No	No
**OUTER MEMBRANE PROTEINS AND LIPIDS**								
TonB dependent receptors	No	Yes^*^	No	Yes^*^	Yes^*^	Yes^*^	No	Yes^*^
OmpA family proteins	No	No	No	Yes^*^	No	No	No	No
Internalin proteins (putative)	No	No	No	Yes^*^	No	No	No	No
Von Willebrand factor type A domain protein	No	No	No	Yes^*^	No	No	No	No
LOS core oligosaccharide biosynthesis	No	No	No	Yes^*^	No	No	No	No
**VITAMIN SYNTHESIS AND TRANSPORT**								
Cobalamin (B12 vitamin) biosynthesis	Yes^*^	No	Yes^*^	No	No	No	No	No
Vitamin B12 ABC transporter	No	No	No	Yes^*^	Yes^*^	Yes^*^	No	Yes^*^
**PHOTOACTIVE PROTEINS**								
Proteorhodopsin	Yes^*^	No	Yes^*^	Yes^*^	Yes^*^	Yes^*^	No	No
Carotenoids	No	No	No	Yes^*^	Yes^*^	Yes^*^	Yes^*^	No

*We also included the transcriptome of Phaeocystis antarctica strain CCMP 1374 (based on 33,153 contigs screened from the assembly of 99,805,691 HiSeq and 2,492,614 pyrosequencing RNA-seq reads). Functional annotation and scenarios were determined using RAST (26)*.

* *Functional assignment made by RAST was manually confirmed using blastx against the nr database in NCBI (blast results are summarized in Dataset S2)*.

** *RAST scenarios, requires the full pathway to be considered detected*.

The capability to assimilate ammonia and glutamate to form glutamine was detected in all the genomes. On the other hand, the anaerobic conversion of nitrate/nitrite to ammonia (denitrification, nitrate respiration) was restricted to the Bacteroidetes, while the Proteobacteria genomes carried genes that encode high affinity transporters for ammonium assimilation. Nitrite/nitrate transporters were detected neither in the *Rhodobacteraceae* nor in the Bacteroidetes genomes while allantoin utilization related genes where detected only in the *Rhodobacteraceae* genome.

The *Rhodobacteraceae* draft genome contained 14 genes related to sulfur oxidation including *soxABRXYZ*, none of which were detected in other draft genomes. This genome (along with that of the putative *Oceanospirillaceae* member) also carried genes required for the conversion of dimethylsulfoniopropionate (DMSP) via DMSP demethylase (DmdA). We also detected two copies of the *dddD* gene (DMSP acyl-CoA transferase) in the SAR92 genome. This gene encodes the conversion to dimethylsulfide (DMS) from DMSP suggesting that in the ASP SAR92 degrades DMSP via a pathway that differs from that present in *Oceanospirillaceae* and *Rhodobacteraceae* related taxa.

Several functions related to carbohydrate metabolism were detected only in the Proteobacteria draft genomes (Table 2). Genes related to carbon monoxide dehydrogenase were detected in the *Rhodobacteraceae* and SAR92 related genomes. Genes related to the pathway of NAD-dependent formate dehydrogenase and pectin degradation were found in the SAR92 genome only. In addition, the SAR92 draft genome harbored several high copy number genes encoding functions related to fatty acid degradation: long-chain-fatty-acid-CoA ligase (*n* = 24), 3-oxoacyl reductase (*n* = 22), enoyl-CoA hydratase (*n* = 14), butyryl-CoA dehydrogenase (*n* = 11) and 3d-ketoacyl-CoA thiolase (*n* = 8). These genes were either not detected or relatively less abundant in other ASP draft genomes. Moreover, these genes were also 2–3 times more abundant when compared to the SAR92 HTCC2207 genome. Genes that encode for glycolate oxidation (especially GlcD) and taurine transport were detected in the *Rhodobacteraceae* and *Oceanospirillaceae* draft genomes.

Genes related to iron scavenging provide information regarding the acquisition strategies for iron among the different microbial taxa. While most draft genomes carried genes for common iron containing molecules like heme, ferredoxin and other Fe-S containing compounds along with those for e.g., ferrochelatase, we found that genes related to siderophore biosynthesis were only present in the Bacteroidetes. Moreover, Fe^3+^ ABC transporter and outer membrane hemin receptors were characteristic to two out of three genomes of free-living Bacteroidetes (*Polaribacter* and *Flavobacteraceae*). In addition, we did not detect TonB dependent receptors used to acquire iron in two Proteobacteria, contrasting with the four other bacterial draft genomes that carry 11–31 of these genes. Finally, the bacterial draft genomes contained different gene complements for Fe uptake: ferric siderophore transport in SAR92, ferric iron ABC transport in the *Oceanospirillaceae, Rhodobacteraceae* and *Cryomorphaceae* and ferrous iron transport protein B in *Polaribacter* and *Flavobacteraceae*.

In addition, these genomes display different genes for mobility (flagella encoding genes in two Proteobacteria vs. genes encoding gliding motility in the three Bacteroidetes) and for outer membrane proteins. In particular, the *Cryomorphaceae* draft genome contained several genes with functions specifically related to outer membranes and lipids. They include cell-to-cell interactions (OmpA, Movva et al., 1980), surface adhesion (von Willebrand factor binding proteins, Hartleib et al., 2000; Cabanes et al., 2002) and internalin surface proteins used as cell wall surface anchors or to invade mammalian cells (Dramsi et al., 1995). They also include Los core oligosaccharides that possess antigenic properties and can act as a barrier to protect bacteria from host-derived antimicrobial compounds (Silipo and Molinaro, 2010). A potential for photoheterotrophy was identified as we detected genes involved in proteorhodopsin (retinal-binding rhodopsin proteins, Béjà et al., 2000) in all draft genomes with exception of that of SAR92. Proteorhodopsins are thought to offer a range of physiological functions to heterotrophic bacteria (Fuhrman et al., 2008), including energy generation from light, suggesting bacteria might harvest a non-negligible fraction of light entering the euphotic zone of Antarctic polynyas. Genes for carotenoid biosynthesis, another photoactive protein involved in protection against oxidative damage, was detected in draft genomes of the three Bacteroidetes and *Micromonas*. Finally, while in some of our draft genomes we detected genes to produce vitamins (B_12_ biosynthesis genes in the *Rhodobacteraceae* and *Oceanopirillaceae* related taxa), in others we found genes to acquire these molecules (vitamin B_12_ ABC transporter detected in the three Bacteroidetes and *P. antarctica*).

### Draft genomes are representative for abundant microbial taxa across the southern ocean

**Figure 5:**
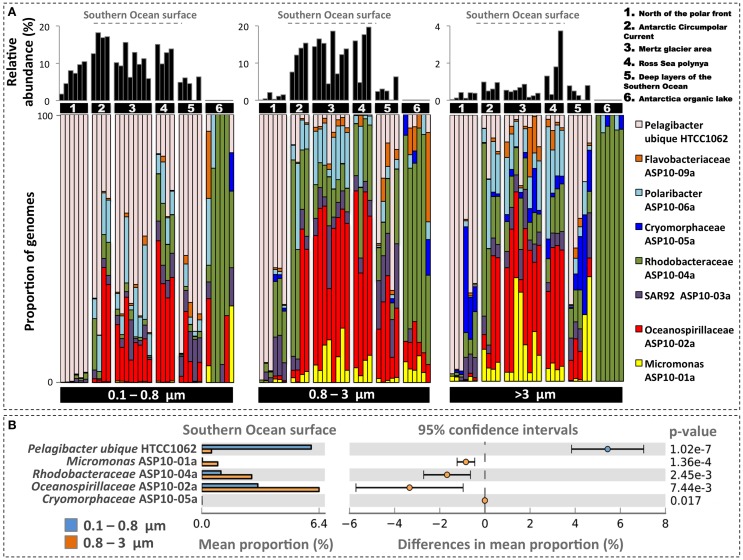
**(A)** Displays the relative abundance for seven draft genomes recovered from the ASP along with the genome of *Pelagibacter ubique* HTCC1062 after mapping to Global Ocean Survey metagenomes. These metagenomes were generated from different locations in the Southern Ocean along with stations north of the polar front (Wilkins et al., 2013a) and in Organic lake, Antarctica (Yau et al., 2013). Colored bars represent the relative proportion (in percentage) of each genome while the black bars represent their relative abundance in the metagenomes. Samples are segregated by size fraction. Samples contained in group 5 were collected at depths below 300 meters. Sample metadata are summarized in Appendix and Dataset S3. **(B)** Displays the mean proportion and distribution of genomes differentially detected between the 0.1–0.8 and the 0.8–3 μm size fractions in Southern Ocean surface samples. Analysis was performed using the *t*-test algorithm contained in STAMP (Parks and Beiko, 2010).

In order to evaluate their ocean basin-wide distribution we mapped metagenome reads of the Global Ocean Survey from multiple locales within and outside the Southern Ocean (Yau et al., 2013; Wilkins et al., 2013a) to the ASP draft genomes. Note that these metagenomes were not used in the assembly and therefore did not contribute to the recovery of the ASP draft genomes. We further mapped these reads to the genome of Candidatus *Pelagibacter ubique* HTCC1062 (a dominant component of the SAR11 population in cold waters, Brown et al., 2012) but did not yield significant assembly in the ASP metagenome analysis. Note that Global Ocean Survey samples were sequenced using pyrosequencing on three size fractions (0.1–0.8 μm, 0.8–3 μm, and >3 μm) and that we used a relatively stringent mapping criterion (>95% sequence identity over the entire read length). The ASP draft genomes were lowly detected north of the polar front and in an Antarctica saline organic lake but they were abundantly present in all Southern Ocean surface stations (with the exception of *Cryomorphaceae* ASP10-5a), indicating a strong latitudinal partitioning (Figure 5A). Thus, we identified the surface waters of the Southern Ocean as the habitat of these taxa. Figure 5B displays the relative contribution of draft genomes across the two smallest size fractions for Southern Ocean surface waters (i.e., stations north of the polar front and of the ice lake were omitted). Five genomes showed significant differences between size fractions. *Oceanospirillaceae* ASP10-02a was the most represented genome (up to 11% of metagenomic reads, consistent with the ASP) and was significantly enriched in the 0.8–3 μm size fraction as compared to the 0.1–0.8 μm size fraction. This genome was also highly detected in a metatranscriptomic dataset generated from the Ross Sea polynya (Bertrand et al., 2015). In contrast *Pelagibacter* reads dominated the 0.1–0.8 μm size fraction. *Micromonas* ASP10-1a, although less abundant, was characteristic for the size fractions >0.8 μm.

## Discussion

Satellite imaging revealed that the bulk of the primary productivity in the Southern Ocean is confined to a few dozen Antarctica polynyas along the continent's coastline (Arrigo and Van Dijken, 2003). Of these the Amundsen Sea Polynya (ASP, West Antarctica) is the most productive per unit of surface (Chlorophyll *a* concentrations peak at ~7 mg/m^−3^, Arrigo and Van Dijken, 2003). Previous studies indicate that the colony-forming haptophyte alga *P. antarctica* is the dominant phytoplankter in the ASP (Alderkamp et al., 2012; Mills et al., 2012; Yager et al., 2012; Kim et al., 2013). ASP microbial communities are enriched with a few specialized bacterial taxa that preferentially associate with *P. antarctica* colonies (Delmont et al., 2014). However, the lack of reference genomes has constrained our ability to analyze functional interactions between bacteria and *P. antarctica* and discern possible roles for heterotrophs in phytoplankton bloom ecology. We determined the metagenome of the ASP phytoplankton-microbial community in the center of the 2010–2011 bloom patch (Yager et al., 2012; Delmont et al., 2014). An abundance of singleton sequences and small contigs (70% of total reads) indicated that genetic structures of *P. antarctica* and diatoms did not assemble efficiently, possibly due to high frequency DNA repeats that are known to dominate large eukaryotic genomes (Richard et al., 2008). The remaining 30% assembled into relatively long genetic structures (scaffolds up to 333 kb). After tetranucleotide frequency clustering, the metagenome assembly efficiently segregated into one eukaryotic (*Micromonas*) and six bacterial (Proteobacteria and Bacteroidetes) draft genomes in which we identified ~42,000 protein coding sequences and more than 5000 different functions. This is the first metagenome report on genome assembly for abundant algal and bacterial taxa in the Southern Ocean. Previous metagenome studies reported on the enrichment of particular bacterial taxa (e.g., Bacteroidetes, Verrucomicrobia, Rhodobacterales) south of the polar front (Wilkins et al., 2013a) and emphasized the capability of *Flavobacteria* in degrading alga-derived organic compounds (Williams et al., 2012; Wilkins et al., 2013b) without attempting *de novo* genome assemblies. Below we discuss a few highlights of the physiological traits and possible ecological functions in the ASP revealed by the draft genomes. Furthermore, the fact that most of these genomes are abundantly present with nucleotide identity of >95% in distant locations (Figure 5) suggests that the functional and ecological traits we describe here for the ASP are representative for the Southern Ocean as a whole.

### High latitude *Micromonas* genome displays features of iron and cold adaptation

The discovery of a pico-eukaryotic *Micromonas* (*Chlorophyta)* genome constitutes an important step toward the analysis of eukaryotic natural populations *in situ*. This draft genome resulted from a highly uniform scaffold cluster (*n* = 1071) with respect to best blast hit taxonomy, %GC and coverage. This draft genome has a size of 19.6 Mb with 22,228 coding sequences which we estimate accounts for roughly 94% of the genomic content of the local *Micromonas* population. *Micromonas* forms only a small but significant fraction of the *Phaeocystis* dominated ASP phytoplankton community and they are even less abundant in offshore samples (Wolf et al., 2013). Members of the *Micromonas* genus occur in a wide range of ecosystems, from the tropics to polar regions (Lovejoy et al., 2007; Foulon et al., 2008). Given the high latitude of the ASP, the new *Micromonas* genome provides an opportunity for study of genome adaptions to extreme environments. It revealed >9000 unique protein coding DNA sequences (CDS identity < 20%) when compared with the genomes of other *Micromonas* and the related *Ostreococcus* (Worden et al., 2009) with an enrichment of genes implicated in fatty acid metabolism. This genome was most similar to that of *Micromonas* strain CCMP1545, but several unique functions indicated cellular adaptation to life at high latitude, including a gene encoding for an anti-freeze protein with no close algal relatives in the NCBI databases. Since only 18% of CDSs were annotated, these trends await further confirmation. Nevertheless, genome adaptation features so far support an earlier hypothesis of a long term glacial refuge for green algae in the Antarctic region (De Wever et al., 2009).

### Bacterial draft genomes represent a significant fraction of the ASP microbial community

Along with the *Micromonas* genome, we recovered nine draft genomes of the dominant bacterial taxa occurring in the bloom (Table 1). The genomes effectively assembled to length of >2.5 Mb are derived from members of the Proteobacteria (SAR92, *Oceanospirillaceae* and *Rhodobacteraceae*) and Bacteroidetes (*Cryomorphaceae, Polaribacter*, and *Flavobacteraceae*). These taxa were identified in various studies as dominant members of the ASP microbial community (Kim et al., 2013; Delmont et al., 2014; Williams et al., 2014). Based on 16S-V6 analyses, these genomes represent about 50% of the bacterial community and >90% of the bacteria associated with *P. antarctica* cells and colonies (Delmont et al., 2014). From a genomic perspective, they comprised 15% of the ASP metagenome with a total of 14,933 protein coding sequences. The protein identity scores of these draft genomes to their nearest relative in the RAST database ranged from 32% (*Cryomorphaceae*) to 69% (*Polaribacter*), emphasizing the valuable addition they make to available genomes as well as highlighting the current lack of reference genomes for the Southern Ocean. From a functional perspective, the draft genomes clustered according to their taxonomy (Proteobacteria vs. Bacteroidetes) rather than their physical association with *Phaeocystis* (free-living vs. particle associated) (Figures 3–4), emphasizing a taxonomy-driven functional compartmentalization. These trends helped us to understand how these microorganisms interact with their environment. For example, the pico-eukaryote genome is enriched in genes related to inorganic sulfur assimilation while the Proteobacteria genomes carry more genes that facilitate the organic assimilation of this element. Below we discuss physiological and potential ecological roles encoded by these draft genomes, with a particular focus on three bacterial groups that were identified as living in close association with *Phaeocystis* colonies in the same bloom (Delmont et al., 2014).

### Antarctic oceanospirillaceae and rhodobacteraceae as sources of cobalamin

B_12_ is produced by Bacteria and Archaea and stimulates algal growth in many aquatic systems (Croft et al., 2005). It also impacts the production of DMSP in the Southern Ocean (Bertrand and Allen, 2012). B_12_ has been implied as a limiting factor for phytoplankton growth in Antarctica polynyas (Bertrand et al., 2007). The occurrence of a dominant, but not yet identified group of B_12_ producing bacteria highlights the knowledge gap in polar systems (Bertrand et al., 2011). *Oceanospirillaceae* related populations are largely underexplored in the Southern Ocean and their link to B_12_ vitamin (and potentially DMSP) production has not been determined prior to this study. The *Oceanospirillaceae* draft genome contains a complete B_12_ vitamin synthesis pathway. Given their dominance in the APS polynya (Kim et al., 2013; Delmont et al., 2014; Williams et al., 2014) and preferential association with *Phaeocystis* colonies, *Oceanospirillaceae* may play an essential role in supporting primary productivity in the Southern Ocean throughout the austral summer. A total of 14 B_12_ vitamin synthesis genes were also found in the *Rhodobacteraceae* draft genome. Several members of this taxon were shown to be B_12_ vitamin producers when associated with phytoplankton at lower latitudes (Wagner-Döbler et al., 2009). Despite their lower abundance and their free-living nature in the ASP (refs), *Rhodobacteraceae* may also play a role in fueling primary production with cobalamin. The remaining bacterial draft genomes had no genes related to this biosynthesis, highlighting the ecological importance of two of the three Proteobacteria taxa that dominate bacterial communities in the ASP. Moreover, the porin TonB is required for cobalamin uptake (Shultis et al., 2006) and its gene was found in all draft genomes but the B_12_ producing *Oceanospirillaceae* and *Rhodobacteraceae*. In a previous publication we highlighted the significance of a near 1:1 cell ratio of SAR92 and algal cells inside *Phaeocystis* colonies (Delmont et al., 2014). The results presented here indicate that *Phaeocystis* does not derive B_12_ from SAR92 and thus this compound does not play a role in in their interactions.

### Proteobacteria related populations can impact sulfur cycle thought DMSP catabolism

A distinct difference between the ASP Proteobacteria and Bacteroidetes is the presence of the gene complement that encodes catabolism of DMSP in the former group. *Phaeocystis* blooms are known to produce copious amounts of DMSP (Gibson et al., 1990; Kirst et al., 1991; DiTullio et al., 2000), and they have a substantial impact on the cycling of sulfur in these systems. DMSP catabolism occurs via two pathways which have different ecological consequences (Kiene et al., 2000; Todd et al., 2007). Here, we determined that abundant members of the *Oceanospirillaceae* and *Rhodobacteraceae* can demethylate DMSP and use this compound as a source of carbon and sulfur. They also possess genes related to glycolate oxidation and taurine transport. Glycolate and taurine are compounds produced by phytoplankton and utilized by specialized heterotrophic bacteria in marine systems (Lau et al., 2007; Amin et al., 2015). The taxonomical link between DMSP/glycolate/taurine catabolism and B_12_ vitamin production suggests a cross-domain functional interaction to enhance the bloom efficiency. Moreover, the ASP SAR92 genome carries two copies of the *dddD* gene that confers the ability to produce the volatile DMS from the cleavage of DMSP. This pathway is of particular interest as DMS has been implicated in cloud formation, and thus constitutes an important climate feedback mechanism (Charlson et al., 1987; Ayers and Gillett, 2000). The contribution of SAR92 to the high DMS concentrations detected in the ASP (Tortell et al., 2012) and other Antarctica polynyas has yet to be determined. However, the tight association of SAR92 with *P. antarctica* colonies during blooms, coupled with the SAR92 capacity to produce DMS, suggests an important role for SAR92 in the Southern Ocean sulfur cycle and an impact on regional climate.

In addition, SAR92 has an extended pool of genes that encode fatty acid catabolism via the beta-oxidation pathway. *Phaeocystis* blooms at other locations were shown to produce large amounts of polyunsaturated fatty acids (Hamm and Rousseau, 2003). In high concentrations fatty acids are toxic to many algae and thus accumulation of fatty acids inside colonies may be problematic for the health of *Phaeocystis* cells. It would therefore be beneficial to the alga if associated bacteria were capable of removing fatty acids. SAR92 has the metabolic machinery to do just that provided that they can take up fatty acids. Although, the ASP SAR92 draft genome lacks the FadL/FadR based fatty acid transport systems, it is highly enriched in TonB dependent transport systems (*n* = 25), which are needed for uptake of—among other substrates—fatty acid linked siderophores. Interestingly, SAR92 was also found in association with phytoplankton blooms elsewhere in the Southern Ocean (West et al., 2008) as well as in the North Sea (Teeling et al., 2012), suggesting an essential role for this organism in bloom ecology.

### *Cryomorphaceae* related population has a unique set of host invasion functions

The *Cryomorphaceae* draft genome was characteristic to the size fraction >3 μm in Southern Ocean stations of the Global Ocean Survey (Figure 5), contrasting with the other bacterial genomes mostly detected in smaller size fractions. In the ASP, *Cryomorphaceae* was the only taxon found in association with *Phaeocystis* colonies both in the photic and aphotic layers of the ASP (Delmont et al., 2014). Members of this family were also found in association with decaying phytoplankton blooms in temperate waters (Pinhassi et al., 2004) and a culture isolate was directly obtained from *P. globosa* colonies (Zhou et al., 2013). The Cyromorphaceae draft genome (along with the two other Bacteroidetes-related organisms) carries genes that encode Fe-scavenging siderophores (Soria-Dengg et al., 2001) beneficial for phytoplankton productivity in the Fe-limited ASP (Martin and Fitzwater, 1988; Martin et al., 1994; Boyd et al., 2007). Increased iron availability inside *P. antarctica* colonies would give *Phaeocystis* a significant advantage over diatoms and other algae. This bacterial association might go a long way in providing an explanation as to why diatoms are more iron limited than *P. antarctica* in an Antarctica polynya (Rose et al., 2009) and so contribute to the success of *P. antarctica* in these systems. In addition, this *Cryomorphaceae* draft genome contains five genes related to cytochrome c551 peroxidase, an enzyme that catalyzes the conversion of hydrogen peroxide to water (Atack and Kelly, 2006). This function was not detected in other genomes (with the exception of one gene in the SAR92 draft genome) and may protect *Phaeocystis* from oxidative stress in an environment with up to 400% oversaturation in oxygen (Yager et al., 2012). On the other hand, across the draft genome collection there were several genes that target cell envelope structures that were only detected in this taxon. Several of these functions are related to virulence and host invasion as observed in pathogenic bacteria like *Listeria, Legionella*, and *Staphylococcus*. This particular functional pool detected only in *Cryomorphaceae*, coupled with its preferential association with *Phaeocystis*, suggests a selective integration inside the colonies and a possible role in *Phaeocystis* decomposition and bloom demise. Future studies should employ spectral imaging (Valm et al., 2011) and controlled *in vitro* experiments to visualize the *Cryomorphaceae*-alga physical interactions and determine the ecological nature and functional consequences of their close association.

### Free-living bacterial populations can also impact the ASP bloom

Essential ecosystem functions were not confined to *Phaeocystis* associated bacterial taxa. Dominant free-living bacterial populations in the polynya can also play a role in the *Phaeocystis* bloom ecology. *Rhodobacteraceae* populations have the capability of oxidizing reduced sulfur compounds to sulfate (*sox*) and so make a crucial contribution to the pelagic sulfur cycle. The *sox* pathway was not detected in any of the other bacterial draft genomes. Free-living Flavobacteriaceae have several genes for iron acquisition that were not found in other taxa. They are also known to degrade high molecular weight, dissolved organic matter produced by algae (Williams et al., 2012; Wilkins et al., 2013b). Lastly, the lack of genome assemblies for the free-living *Pelagibacter*, an abundant member of the ASP bacterial community, suggests that this taxon harbors a high degree of diversity and possibly low synteny among its members and it thus warrants a targeted genome study.

## Materials and methods

### Sampling

Water samples were taken with a CTD Rosette equipped during a phytoplankton bloom event in the ASP in 2010–2011. Geochemical characteristics, *Phaeocystis* physiology, heterotrophic activity and bacterial community structures of these samples have been reported previously (Yager et al., 2012; Delmont et al., 2014; Williams et al., 2014). For our metagenomic investigation we selected DNA from a surface layer sample that was collected in the center of the bloom (073° 34′243S 112° 40′080W, chlorophyll *a* > 17 μg/L, temperature of −1.2°C, phosphate: 1.31 μM, nitrite: 0.02 μM, ammonium: 0.05 μM, silicate: 77.8 μM) on 19 December 2010. This sample (6 l, 10 m depth) was passed over a 20 μm mesh, collected onto a 0.2 μm Sterivex membrane filter cartridge by pressure filtration, quickly frozen in the headspace of a LN_2_ dewar and stored at −80°C. DNA extraction was performed using the Puregene kit (Gentra) after disruption of the cells with lytic enzyme coupled to proteinase K (Sinigalliano et al., 2007). DNA was quantified using a Nanodrop 2000 instrument (Thermo Fisher Scientific, Wilmington, DE).

### DNA sequencing

We subsequently generated metagenomic libraries with the OVATION ultralow kit (NuGen) using 100 ng of DNA and 8 amplification cycles. We constructed overlapping (2X100 nt with ~40 nt of overlap) and gapped (2X108 nt with an insert size of ~600 nt) metagenomic DNA libraries using a Pippin prep electrophoresis platform to precisely select the desired length for DNA fragments to be used for sequencing on a Hiseq platform (Illumina). For our gapped reads we used “analyze-illumina-quality-minoche” script that implements the quality filtering approach (Minoche et al., 2011). For our overlapping reads we used “merge-illumina-pairs script” script with default parameters (0 mismatches allowed), which relies on the overlapping region for quality filtering while merging paired-end reads (Eren et al., 2013). Web address http://github.com/meren/illumina-utils gives access to both scripts. The total size of our final quality-filtered dataset was 23 Gb with 159,277,396 sequences. Gapped metagenomic library consisted 88.14% of our final reads. Quality controlled overlapping reads are publically available on MG-RAST (http://metagenomics.anl.gov/) under project ASPIRE and accession number 4520502.3.

### Metagenome assembly and analysis

First, we annotated our overlapping reads (~160 nt) using MG-RAST (Meyer et al., 2008) to evaluate the overall diversity and functionality of the polynya surface using various reference databases. Then, we used CLC (version 6) to assemble our short reads into larger scaffolds and for mapping. We required a minimum of 97% sequence identity over the full-length of short reads for both the assembly and coverage estimation steps. We analyzed the tetranucleotide frequencies of our scaffolds using an in-house script written in R (Ihaka and Gentleman, 1996). We then used hierarchical clustering (hclust function in stats package for R with Euclidean distance as distance metric) to order our scaffolds based on their tetranucleotide frequency profiles. We generated our draft genomes by binning scaffolds clustered together in well-supported clades represented in the resulting tree. To reduce heterogeneity in our genome bins we also used coverage, GC-content and taxonomy (phymmBL, Brady and Salzberg, 2009 with an *e*-value cut-off of 10^−5^) to define our contig clusters. For better taxonomic classification, we increased the resolution of phymmBL by adding the genomes of *Pelagibacter ubique* (HTCC1062), *Marinomonas* (MED121), *Micromonas pussila* (CCMP1545), *Phaeodactylum tricornutum* (CCAP10551), *Polaribacter franzmannii* (ATCC700399), *Polaribacter irgensii* (23-P), *SAR92* (HTCC2207), and *Thalassiosira pseudonana* (CCMP1335). Finally, we removed eukaryotic chloroplast and mitochondria genetic fragments for downstream bacterial genome analyses (these scaffolds were clustered with the bacterial scaffolds due to a more similar evolutionary origin, Douglas, 1998). Note that our metagenomic assembly visualization and binning approaches can be performed using the anvi'o platform (Eren et al., 2015).

### Genome analysis

We used RAST (Aziz et al., 2008) and FIGfam (Meyer et al., 2009) version 64 to annotate our scaffolds. We then compared our draft genomes to available genomes at the protein level in RAST. When performing these comparisons, protein identity scores ranged from 100% when identical to 0% when detected only in our draft genomes. Principal component analyses were performed to compare the functional potential of these draft genomes using R software and the Ade4TkGUI package (Thioulouse et al., 1997). For statistics underpinnings we applied *t*-test, embedded within STAMP (Parks and Beiko, 2010) to ascertain significance to distribution of functions across genome clusters as well as on relative genome abundances in different metagenome size fractions. Finally, we used Gephi v0.8.2 (Bastian et al., 2009) to generate functional networks (Force Atlas 2) connecting different collections of genomes. Draft genomes (and the proteins they encode for) have been deposited in http://dx.doi.org/10.6084/m9.figshare.1320614 and as NCBI project PRJNA276743 (http://www.ncbi.nlm.nih.gov/bioproject/PRJNA276743). PRJNA276743 also provides proteins and their functions as inferred by NCBI.

### Statement of significance

Massive phytoplankton blooms in climate-sensitive Antarctic polynyas are an ecologically important phenomenon but the functional underpinnings of their intensity and persistence are not entirely understood. *Phaeocystis antarctica* blooms sustain a distinct bacterial community that was hypothesized to play a role in bloom duration and intensity. The metagenome of a *P. antarctica* bloom in the Amundsen Sea polynya yielded draft genomes of multiple bacterial taxa, some of which are known to associate with *Phaeocystis* colonies. The analysis of functional complementarity between *P. antarctica* and these bacterial taxa revealed essential roles for these taxa in Fe scavenging, vitamin B_12_ production, fatty acid consumption and sulfur metabolism and contribute significantly to our understanding of phytoplankton bloom ecology in the Southern Ocean.

### Conflict of interest statement

The authors declare that the research was conducted in the absence of any commercial or financial relationships that could be construed as a potential conflict of interest.

## Supplementary material

Figure S1 **Relative abundance of domains of life (A) and most abundant phyla (B) detected in our overlapping metagenomic data depending on the reference database used (using proteins vs. rRNA genes) in MG-RAST (Meyer et al., 2008)**. Note that the underrepresentation of Eukaryota when using protein databases is probably due to the current lack of eukaryotic genomes available.

Figure S2 **Hierarchical clustering (Euclidean distance metric) based on tetranucleotide frequency profiles of the 371 scaffolds representing the cluster 2 of Figure 1**. Informative layers were added below to the clustering tree. Taxonomical affiliation was inferred using phymmBL (Brady and Salzberg, 2009).

Figure S3 **Hierarchical clustering (Euclidean distance metric) of 848 scaffolds (>5 kb in length) based on their tetranucleotide frequency profiles**. Scaffolds were assembled using 10 million gapped reads to optimize the recovery of the most dominant genetic structures. Four informative layers were added below to the clustering tree. Taxonomical affiliation was inferred using phymmBL (Brady and Salzberg, 2009).

Figure S4 **Hierarchical clustering (Euclidean distance metric) of 1000 scaffolds (>6.7 kb in length) based on their tetranucleotide frequency profiles**. Scaffolds were assembled using 20 million gapped reads to optimize the recovery of the dominant and sub-dominant genetic structures. Four informative layers were added below to the clustering tree. Taxonomical affiliation was inferred using phymmBL (Brady and Salzberg, 2009).

Figure S5 **Hierarchical clustering (Euclidean distance metric) of 1000 scaffolds (>10.1 kb in length) based on their tetranucleotide frequency profiles**. Scaffolds were assembled using 40 million gapped reads to optimize the recovery of the dominant and sub-dominant genetic structures. Four informative layers were added below to the clustering tree. Taxonomical affiliation was inferred using phymmBL (Brady and Salzberg, 2009).

Figure S6 **Hierarchical clustering (Euclidean distance metric) of 3553 scaffolds (>6 kb in length) based on their tetranucleotide frequency profiles**. Scaffolds were assembled using the entire metagenomic dataset to optimize the recovery of the less abundant genetic structures. Five informative layers were added below to the clustering tree. Taxonomical affiliation was inferred using phymmBL (Brady and Salzberg, 2009). A cluster of 1071 scaffolds affiliated to *Micromonas* and representing a total of 19.6 Mb was recovered from this analysis.

Figure S7 **Length of scaffolds associated to the *Micromonas* ASP10-1a draft genome**. A scaffold length logarithmic regression curve was performed (*R*^2^ > 0.997). A total of 437 scaffolds smaller than 6 kb (representing 1.3 Mb) are estimated to be missing from the draft genome. Note that the completion score of 93.8% is only based on this scaffolds length regression curve.

Figure S8 **Network connecting the *Micromonas* ASP10-1a draft genomes recovered from the Amundsen polynya plus four Chlorophyta genomes and their annotated functions from RAST (Aziz et al., 2008) (a total of 3.086 functions)**. The network was performed using Gephi (Bastian et al., 2009) and Force Atlas 2.

Dataset S1 **Statistical analyses (*t*-test and equal variance, *p*-value cut-off of 0.05) of functions detected in the six bacterial draft genomes (*n* = 4253) in order to discriminate functions differentially present between (I) taxa (Bacteroidetes vs. Proteobacteria) and (II) life styles [free-living taxa (Rhodobacteraceae; Polaribacter; Flavobacteriaceae) and particle-associated taxa (Oceanospirillaceae; SAR92; Cryomorphaceae)]**.

Dataset S2 **Summary of online blastx against nr (NCBI database) for 86 genes of particular interest in the study**. RAST ORFs can be found in the Genbank files. Note that only the first best hit matching the RAST functional annotation is summarized. All *e*-values were below e-10.

Dataset S3 **Metadata summary for the Global Ocean Survey stations utilized in Figure 5**.

## Appendix

### Supplemental clusters information

We first ordered the 1500 longest scaffolds (from the primary assembly) using tetranucleotide frequencies (Figure 1). Most scaffolds were ordered in clusters that possess a stable GC content and inferred taxonomy, emphasizing the strength of tetranucleotide frequency binning genetic structures longer than 10 kb into biologically relevant draft genomes.

Cluster 1: The first cluster is represented by 660 scaffolds for which cumulated length, coverage and GC content reach 16 Mb, 50.3 ± 3.8X and 58.8 ± 1.5%, respectively. 74.5% of scaffolds present in this cluster are related to a *Micromonas pussilla* genome (*Chlorophyta* phylum). Moreover, the high percentage of hypothetical proteins in these scaffolds when annotated with RAST (81.9 ± 10.4%) suggests a eukaryotic origin. Moreover, small GC content variations can be observed between the sub-groups of this cluster in spite of a constant coverage, suggesting the presence of different chromosomes belonging to the same organism.

Cluster 2: The second cluster (8.9 Mb in length) displays low GC content scaffolds (30.6 ± 1.8%). Moreover, 94.1% and 81.9% of these 376 scaffolds were affiliated to the *Flavobacteraceae* family and the *Polaribacter* genus, respectively. Interestingly, at least three chloroplast genomes (including *Phaeocystis* chloroplast represented by two scaffolds of 43 and 44 kb) clustered with this bacterial taxonomic group. However, these biological outliers were easily detected using taxonomical annotation and coverage discrepancies (reaching up to 675X for *Phaeocystis*).

Cluster 3: The third cluster possesses 302 scaffolds for a total length of 7Mbp and a GC content of 43.8 ± 3.0%. But while 93.9% of the annotated scaffolds are affiliated to the Proteobacteria phylum, the cluster can be divided into three distinct groups affiliated to *Oceanopirillaceae, Rhodobacteraceae* and the *SAR92* genus. The *Oceanopirillaceae* cluster is only 1.9Mbp in length with coverage of 348.5 ± 224X. Less abundant, the *Rhodobacteraceae* and *SAR92* clusters have a length of 2.8 and 3.2 Mb and coverage values of 73.2 ± 19.7X and 61.0 ± 49.1X, respectively.

Cluster 4: Contrasting with the three other clusters clearly dominated by *Chlorophyta, Bacteroidetes*, and *Proteobacteria* genomic fragments, the fourth cluster (5Mbp) is a mix of different eukaryotic chloroplasts and mitochondrial fragments (GC content of 55.5 ± 5.5%) along with a well assembled group (44 scaffolds, 2.9 Mb, coverage of 63.7 ± 16.3X, GC content of 37.9 ± 0.9%) affiliated to *Flavobacteraceae* and *Cryomorphaceae*. Finally, two small groups affiliated to the Bacteroidetes and Proteobacteria phyla are present in the extreme right of the Figure 1, but possess lengths of only 453 kb and 280 Kb, respectively. They were subsequently not further investigated in the present study.

### Refining the assembly of high coverage bacterial genomes

We optimized the recovery of genomes using three approaches. First, we generated a tetranucleotide frequency tree based on contigs affiliated to the Cluster 2 only to optimize the partitioning of closely related genomes affiliated to Polaribacter (Figure S2, see Section Refining the Binning of Genomes from Cluster 2). Second, we assembled sub-samples of the metagenomic dataset (10, 20, and 40 million gapped pair reads) to optimize assembly of the most dominant bacterial genomes (see Figures S3–S5). In fact, some bacterial genomes were fragmented in part due to the extent of their coverage values (e.g., up to 743X for the *Oceanospirillum* cluster) (see Section Refining the Assembly of High Coverage Bacterial Genomes). Finally, we found that while the tetranucleotide frequency clustering gains in sensitivity with scaffolds length, this sensitivity was taxa dependent and can be optimize manually. Therefore, we applied different scaffolds length cut-offs depending on the targeted taxonomical group (>11 kb for the bacteria, >6 kb for the eukaryotes) to optimize genomic recovery. A gain of 3.6M in the eukaryotic genomes was possible using this strategy (Figure S6, see Section Optimizing the Chlorophyta Genome Completion).

#### Refining the binning of genomes from cluster 2

We ordered specifically cluster 2 using the same ordination approach as in Figure 1. Scaffolds were organized into five distinct groups for which taxonomical trends can be observed at the species level (Figure S3). In particular, while the high coverage group [196.8X (±57.8) and 1.3 Mb in length] is mostly related to *Polaribacter irgensii* (96.8% of the scaffolds), this species is affiliated to only 25.7% of the scaffolds present in the 4 other groups, supporting the reliability of the different clusters in spite of an important taxonomical similarity.

#### Refining the assembly of high coverage bacterial genomes

While the *SAR92, Rhodobacteraceae*, and *Cryomorphaceae* related genomes appear to be relatively well recovered from our first metagenomic assembly effort, genomes related to *Oceanopirillaceae* and the dominant *Polaribacter* were poorly recovered, partly due to a too important coverage when using the entire dataset. We sequentially assembled 10, 20, and 40 millions of unmerged pair reads as an effort optimizing their assembly, (see Figures S3–S5). Contrasting with the primary assembly, we gained 0.6 Mb of genomic content for the *Oceanopirillaceae* when using a sub-sample of 10 million pair reads (Figure S3). Furthermore, the *Polaribacter irgensii* cluster gained 1.6 Mb when using 20 million pair reads in comparison to what was recovered from the primary assembly (Figure S4). Note that we didn't gain any additional genomic content when using 40 million pair reads (see Figure S5).

#### Optimizing the chlorophyta genome completion

The distinct tetranucleotide frequency of genetic fragments related to the Chlorophyta genome in comparison to the bacteria (Figure 1) provided an opportunity decreasing the length cut-off of scaffolds that can be efficiently ordered. When reducing this cut-off to 6 kb, a total of 3553 scaffolds can be ordered and analyzed, providing a total gain of 17.6Mbp in comparison to the first 1500 scaffolds. The 2053 additional scaffolds provided substantial noise in the analysis of the bacterial genomes (e.g., a Gammaproteobacteria group located within the Bacteroidetes cluster). On the other hand, the Chlorophyta cluster was still highly stable with a genomic content gain of 3.6 Mb (Figure S6), therefore reaching 19.6Mbp with 1071 scaffolds.

### Confirmation of RAST annotation for key functional genes

In order to confirm the RAST annotation of draft genomes, we performed blastx analysis for 95 key genes described in the study against the *nr* database in NCBI. We so confirmed the functional inference for 86 of the 95 genes. The 9 unconfirmed gene functions were omitted from further analyses. Blast results are summarized in Dataset S2.

### Order of the global ocean survey stations displayed in figure 5

Global Ocean Survey stations selected for the geographic locations in Figure 5 are (from left to right): 1- stations 368, 367, 366, 346, 364, and 363; 2- stations 358, 362, 359, and 357; 3- stations 352, 351, 360, 353, 355, 347, 349, and 348; 4- stations 369, 370, 371, and 372; 5- stations 354, 356, 361, 365, and 350; 6- stations 374, 375, 376, 377, 378, and 379. Metadata for these stations has been summarized in Dataset S3.
