# Supplementary figures and images for: Genome reconstructions indicate the partitioning of ecological functions inside a phytoplankton bloom in the Amundsen Sea, Antarctica
Source: Front Microbiol. 2015 Oct 26;6:1090. doi: 10.3389/fmicb.2015.01090 (PMC4620155; doi:10.3389/fmicb.2015.01090)

# Metagenome

V6  
Amplicon

Domains relative  
abundance (%)

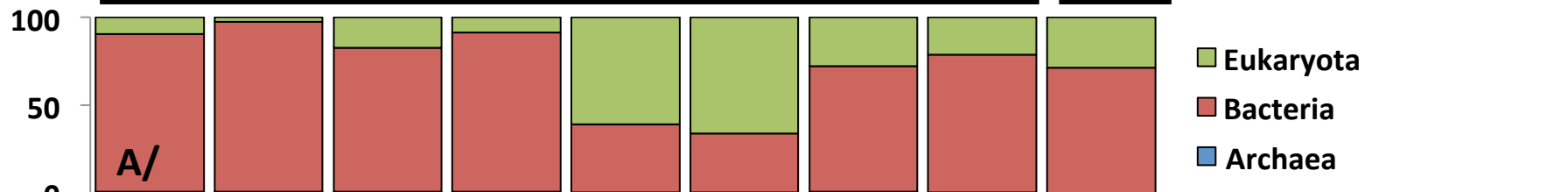

Phylum relative  
abundance (%)

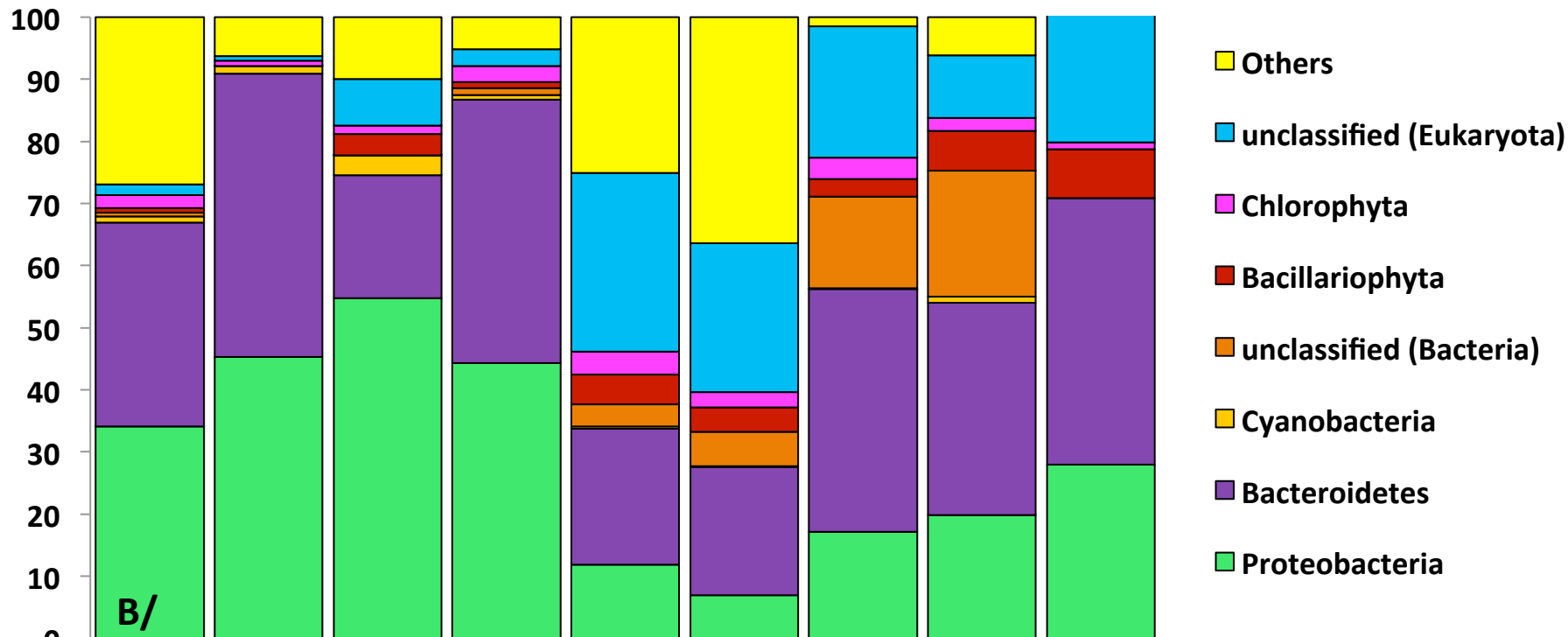

Proteins

RNA

Supplement: Figure S1 — Relative abundance of domains of life (A) and most abundant phyla (B) detected in our overlapping metagenomic data depending on the reference database used (using proteins vs. rRNA genes) in MG-RAST (Meyer et al., 2008). Note that the underrepresentation of Eukaryota when using protein databases is probably due to the current lack of eukaryotic genomes available. [file FigureS1.PDF]

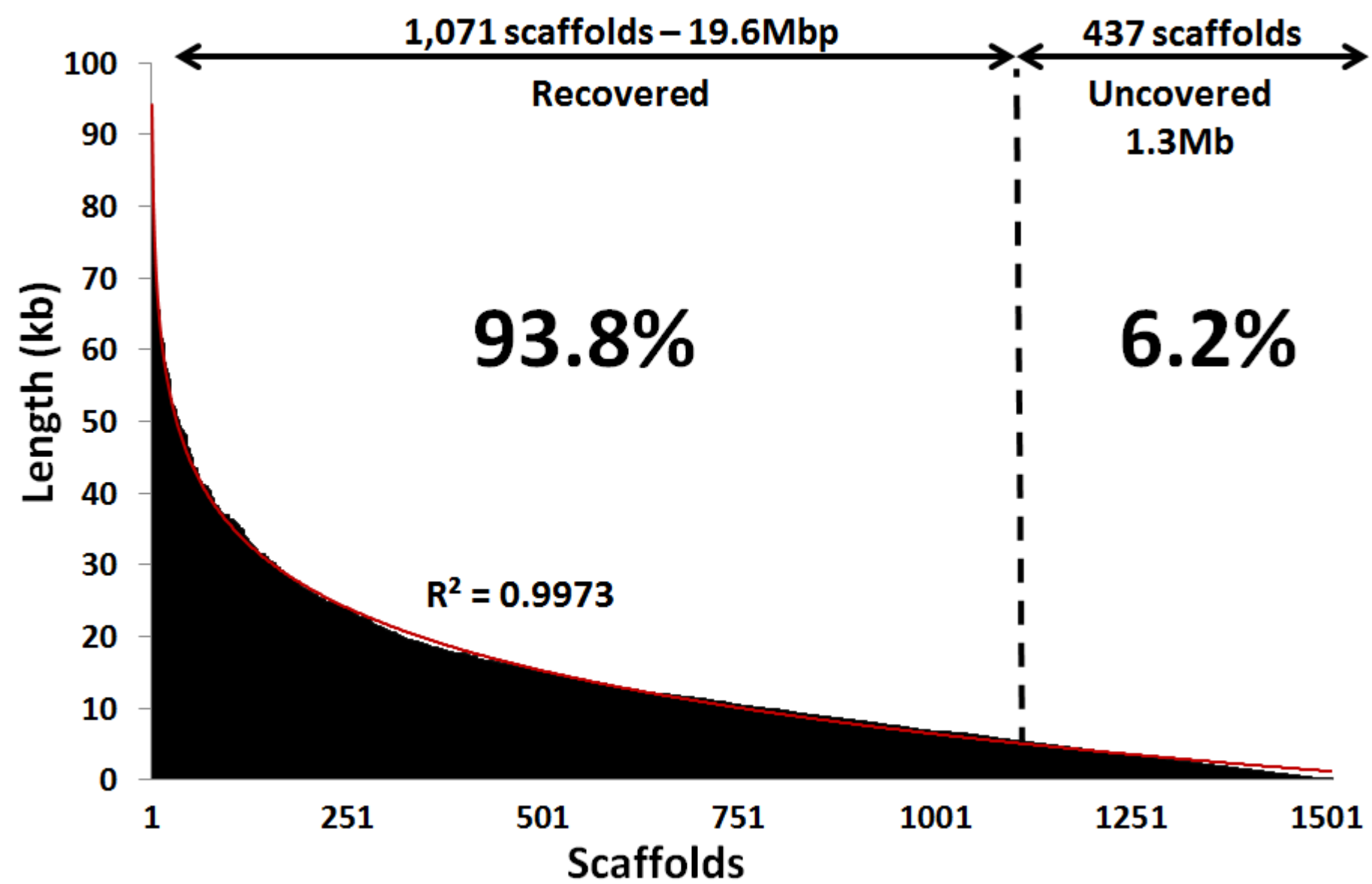

Supplement: Figure S7 — Length of scaffolds associated to the Micromonas ASP10-1a draft genome. A scaffold length logarithmic regression curve was performed (R2 > 0.997). A total of 437 scaffolds smaller than 6 kb (representing 1.3 Mb) are estimated to be missing from the draft genome. Note that the completion score of 93.8% is only based on this scaffolds length regression curve. [file FigureS7.PDF]

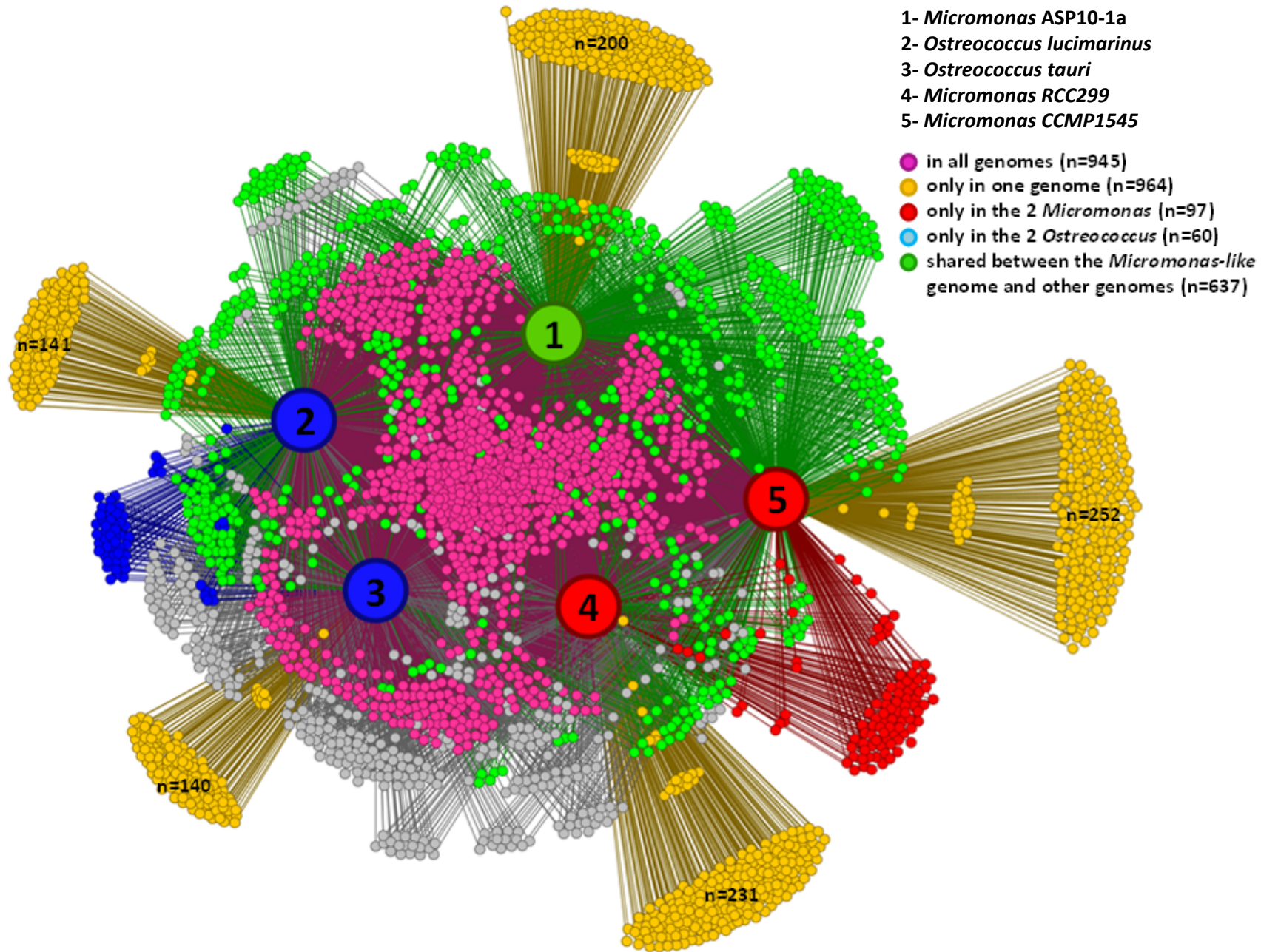

Supplement: Figure S8 — Network connecting the Micromonas ASP10-1a draft genomes recovered from the Amundsen polynya plus four Chlorophyta genomes and their annotated functions from RAST (Aziz et al., 2008) (a total of 3.086 functions). The network was performed using Gephi (Bastian et al., 2009) and Force Atlas 2. [file FigureS8.PDF]
