# Supplementary material for: Genome reconstructions indicate the partitioning of ecological functions inside a phytoplankton bloom in the Amundsen Sea, Antarctica
Source: Front Microbiol. 2015 Oct 26;6:1090. doi: 10.3389/fmicb.2015.01090 (PMC4620155; doi:10.3389/fmicb.2015.01090)
Supplement: Figure S2 — Hierarchical clustering (Euclidean distance metric) based on tetranucleotide frequency profiles of the 371 scaffolds representing the cluster 2 of Figure 1. Informative layers were added below to the clustering tree. Taxonomical affiliation was inferred using phymmBL (Brady and Salzberg, 2009). [file FigureS2.PDF]

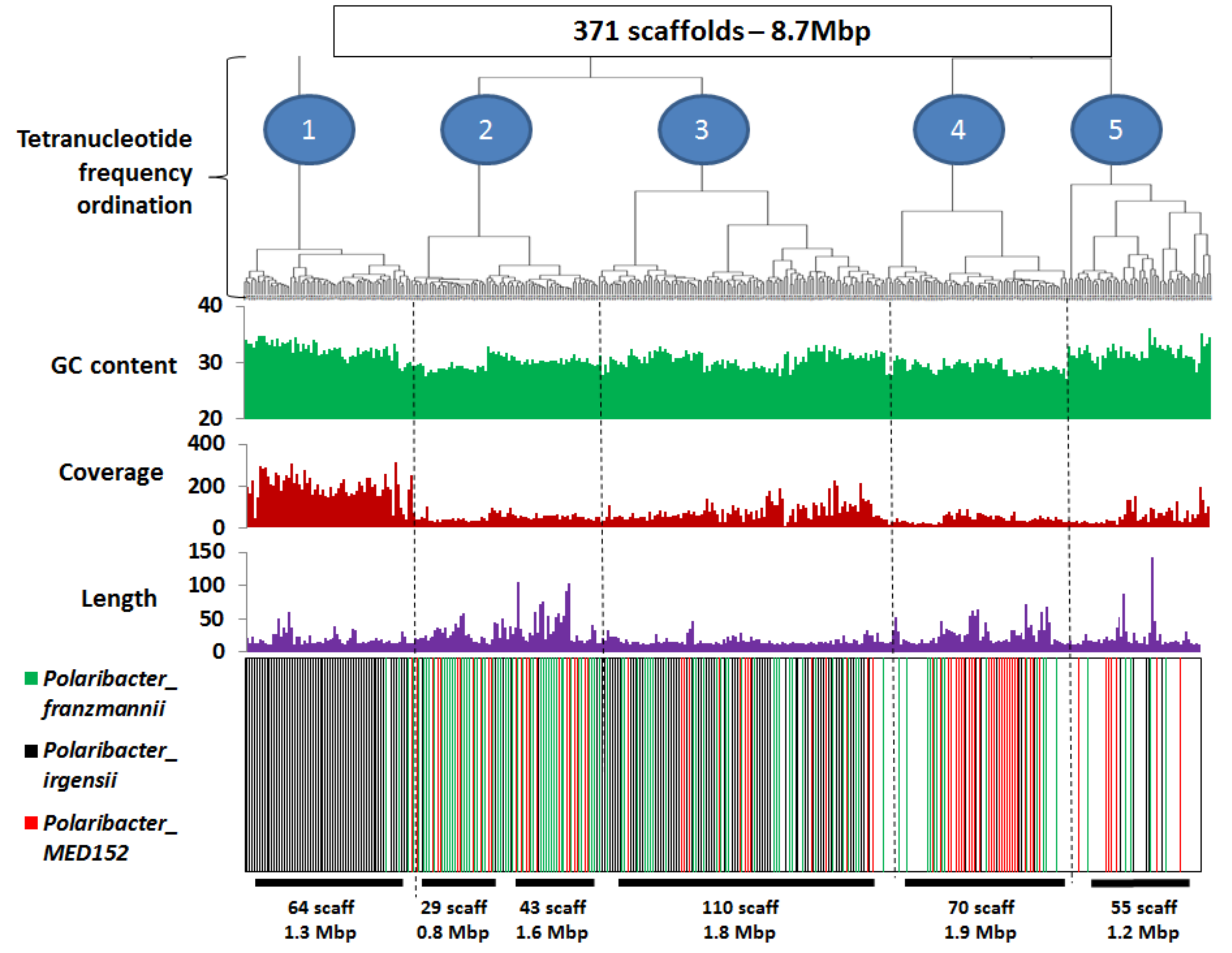

371 scaffolds – 8.7Mbp
Tetranucleotide frequency ordination
1
2
3
4
5
GC content
40
30
20
Coverage
400
200
0
Length
150
100
50
0
Polaribacter_franzmannii
Polaribacter_irgensii
Polaribacter_MED152
64 scaff
1.3 Mbp
29 scaff
0.8 Mbp
43 scaff
1.6 Mbp
110 scaff
1.8 Mbp
70 scaff
1.9 Mbp
55 scaff
1.2 Mbp
