# Supplementary material for: Genome reconstructions indicate the partitioning of ecological functions inside a phytoplankton bloom in the Amundsen Sea, Antarctica
Source: Front Microbiol. 2015 Oct 26;6:1090. doi: 10.3389/fmicb.2015.01090 (PMC4620155; doi:10.3389/fmicb.2015.01090)
Supplement: Figure S3 — Hierarchical clustering (Euclidean distance metric) of 848 scaffolds (>5 kb in length) based on their tetranucleotide frequency profiles. Scaffolds were assembled using 10 million gapped reads to optimize the recovery of the most dominant genetic structures. Four informative layers were added below to the clustering tree. Taxonomical affiliation was inferred using phymmBL (Brady and Salzberg, 2009). [file FigureS3.PDF]

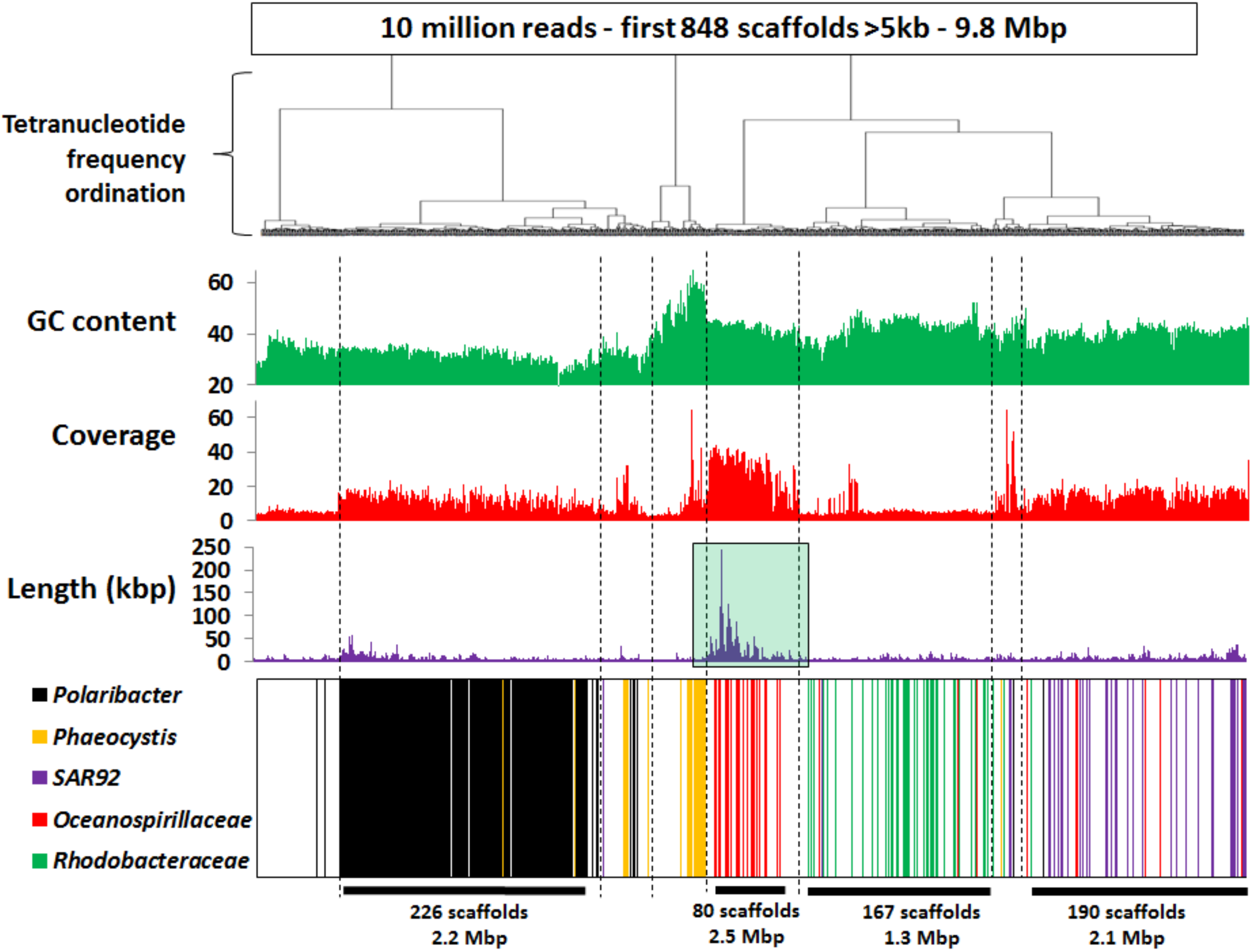

10 million reads - first 848 scaffolds >5kb - 9.8 Mbp
Tetranucleotide frequency ordination
GC content
60
40
20
Coverage
60
40
20
0
Length (kbp)
250
200
150
100
50
0
Polaribacter
Phaeocystis
SAR92
Oceanospirillaceae
Rhodobacteraceae
226 scaffolds
2.2 Mbp
80 scaffolds
2.5 Mbp
167 scaffolds
1.3 Mbp
190 scaffolds
2.1 Mbp
