# Supplementary material for: Genome reconstructions indicate the partitioning of ecological functions inside a phytoplankton bloom in the Amundsen Sea, Antarctica
Source: Front Microbiol. 2015 Oct 26;6:1090. doi: 10.3389/fmicb.2015.01090 (PMC4620155; doi:10.3389/fmicb.2015.01090)
Supplement: Figure S4 — Hierarchical clustering (Euclidean distance metric) of 1000 scaffolds (>6.7 kb in length) based on their tetranucleotide frequency profiles. Scaffolds were assembled using 20 million gapped reads to optimize the recovery of the dominant and sub-dominant genetic structures. Four informative layers were added below to the clustering tree. Taxonomical affiliation was inferred using phymmBL (Brady and Salzberg, 2009). [file FigureS4.PDF]

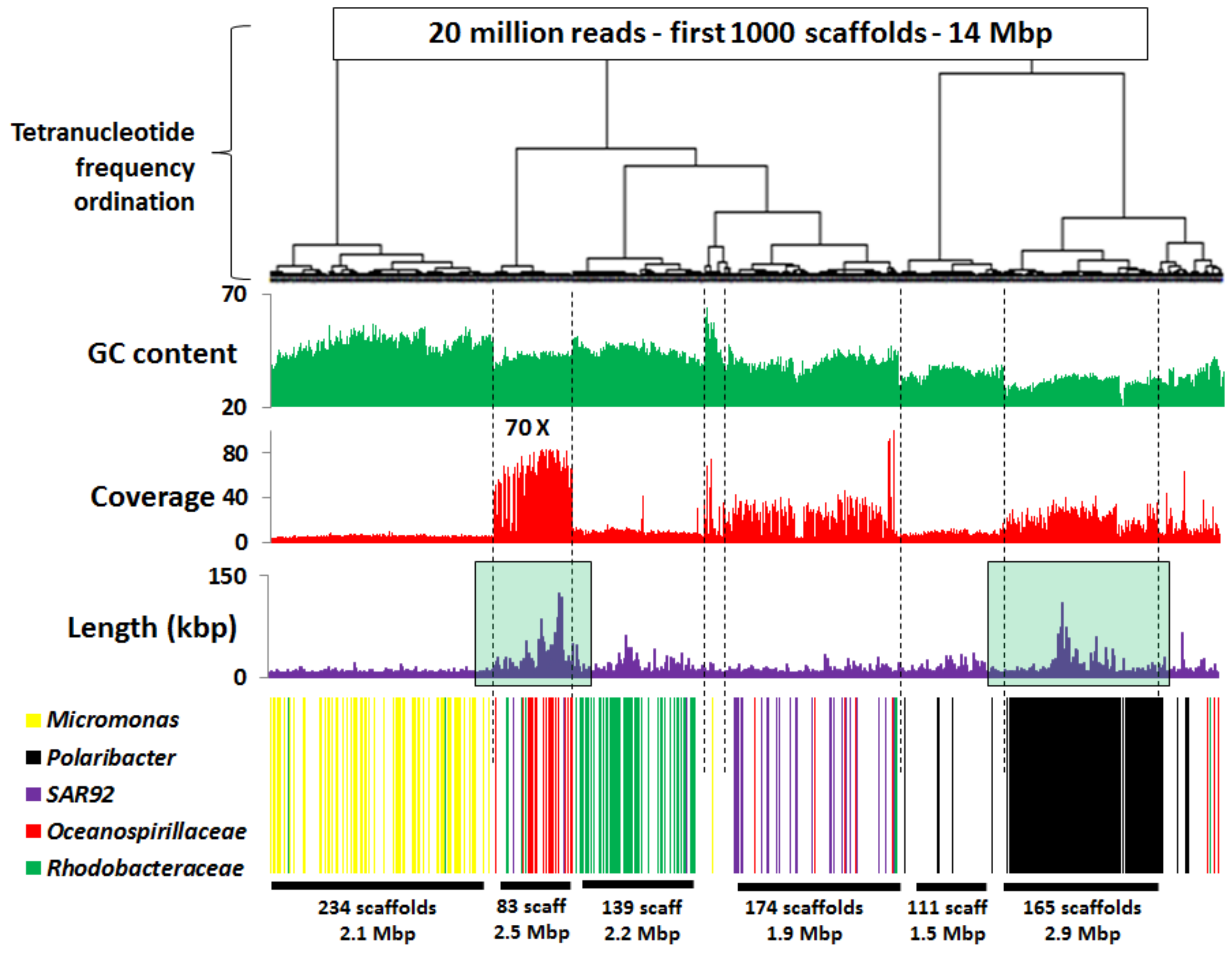
20 million reads - first 1000 scaffolds - 14 Mbp
Tetranucleotide frequency ordination
GC content
70
20
70 X
Coverage
80
40
0
Length (kbp)
150
0
Micromonas
Polaribacter
SAR92
Oceanospirillaceae
Rhodobacteraceae
234 scaffolds 2.1 Mbp
83 scaff 2.5 Mbp
139 scaff 2.2 Mbp
174 scaffolds 1.9 Mbp
111 scaff 1.5 Mbp
165 scaffolds 2.9 Mbp
