# Supplementary material for: Genome reconstructions indicate the partitioning of ecological functions inside a phytoplankton bloom in the Amundsen Sea, Antarctica
Source: Front Microbiol. 2015 Oct 26;6:1090. doi: 10.3389/fmicb.2015.01090 (PMC4620155; doi:10.3389/fmicb.2015.01090)
Supplement: Figure S5 — Hierarchical clustering (Euclidean distance metric) of 1000 scaffolds (>10.1 kb in length) based on their tetranucleotide frequency profiles. Scaffolds were assembled using 40 million gapped reads to optimize the recovery of the dominant and sub-dominant genetic structures. Four informative layers were added below to the clustering tree. Taxonomical affiliation was inferred using phymmBL (Brady and Salzberg, 2009). [file FigureS5.PDF]

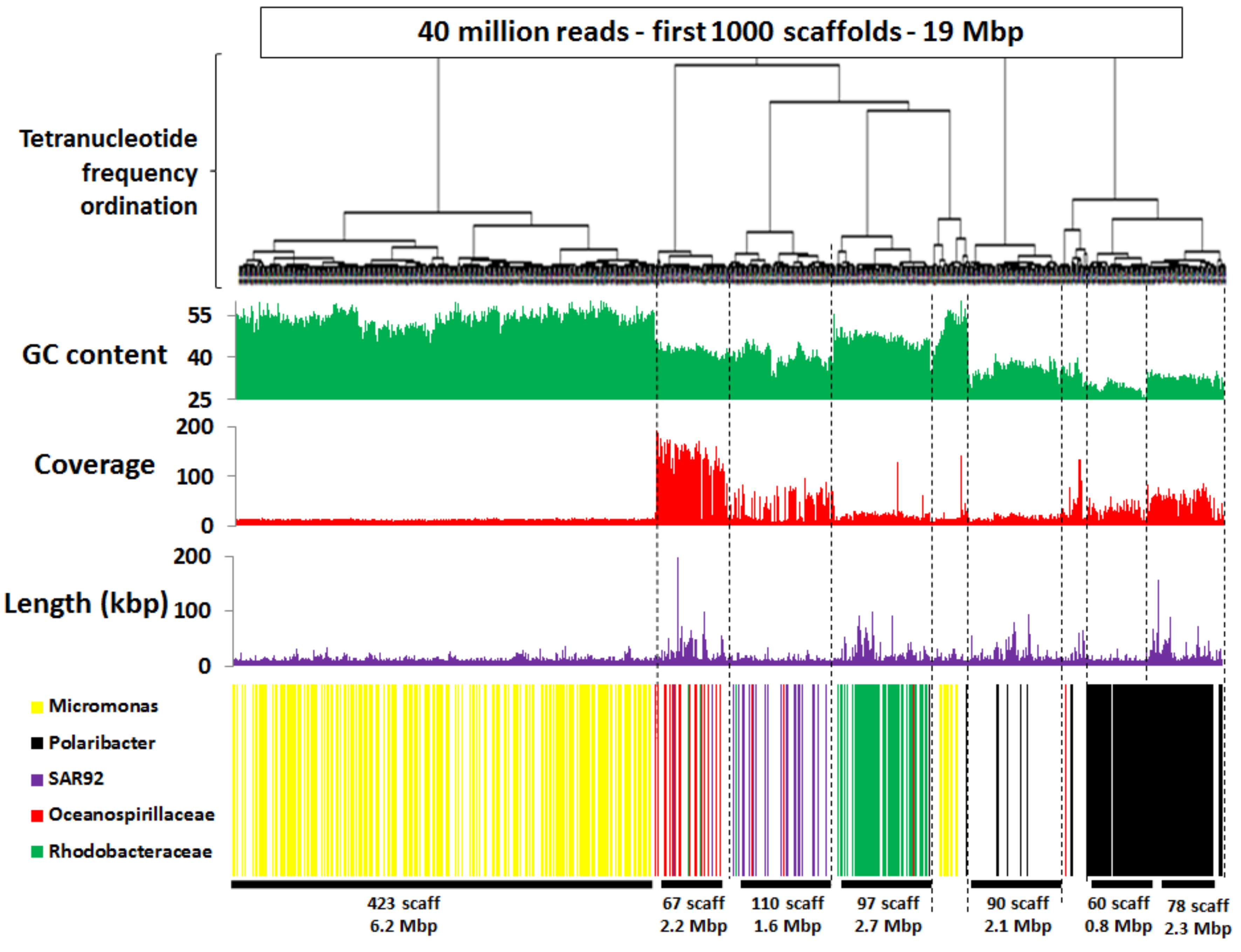
40 million reads - first 1000 scaffolds - 19 Mbp
Tetranucleotide frequency ordination
GC content
55
40
25
Coverage
200
100
0
Length (kbp)
200
100
0
Micromonas
Polaribacter
SAR92
Oceanospirillaceae
Rhodobacteraceae
423 scaff
6.2 Mbp
67 scaff
2.2 Mbp
110 scaff
1.6 Mbp
97 scaff
2.7 Mbp
90 scaff
2.1 Mbp
60 scaff
0.8 Mbp
78 scaff
2.3 Mbp
