# Supplementary material for: Genome reconstructions indicate the partitioning of ecological functions inside a phytoplankton bloom in the Amundsen Sea, Antarctica
Source: Front Microbiol. 2015 Oct 26;6:1090. doi: 10.3389/fmicb.2015.01090 (PMC4620155; doi:10.3389/fmicb.2015.01090)
Supplement: Figure S6 — Hierarchical clustering (Euclidean distance metric) of 3553 scaffolds (>6 kb in length) based on their tetranucleotide frequency profiles. Scaffolds were assembled using the entire metagenomic dataset to optimize the recovery of the less abundant genetic structures. Five informative layers were added below to the clustering tree. Taxonomical affiliation was inferred using phymmBL (Brady and Salzberg, 2009). A cluster of 1071 scaffolds affiliated to Micromonas and representing a total of 19.6 Mb was recovered from this analysis. [file FigureS6.PDF]

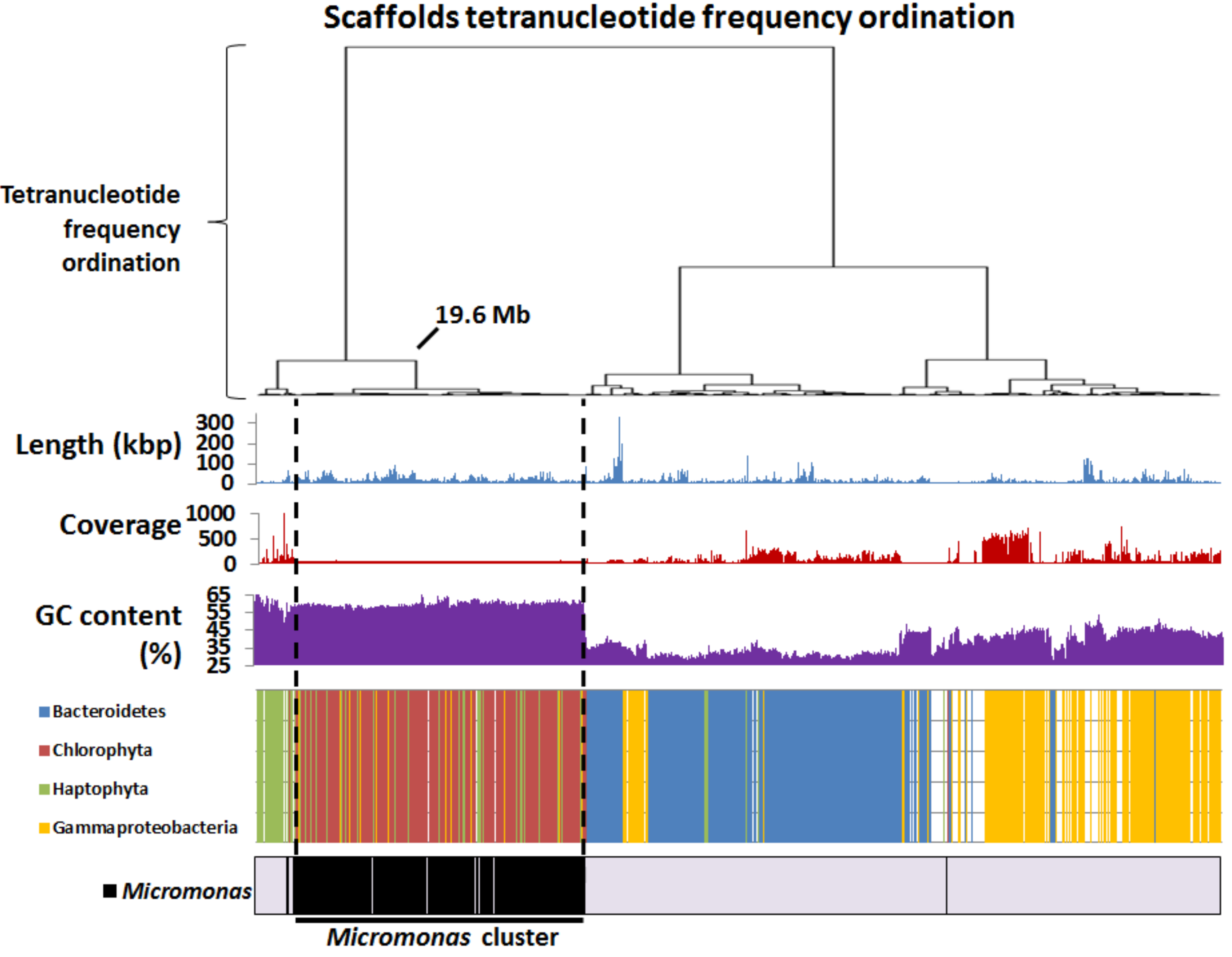

Scaffolds tetranucleotide frequency ordination
Tetranucleotide frequency ordination
19.6 Mb
Length (kbp)
300
200
100
0
Coverage
1000
500
0
GC content (%)
65
55
45
35
25
Bacteroidetes
Chlorophyta
Haptophyta
Gammaproteobacteria
Micromonas
Micromonas cluster
